# Volatile Memristive Devices with Analog Resistance Switching Based on Self-Assembled Squaraine Microtubes as Synaptic Emulators

**DOI:** 10.1021/acsami.3c13735

**Published:** 2024-01-04

**Authors:** Karl Griffin, Gareth Redmond

**Affiliations:** School of Chemistry, University College Dublin, Belfield, Dublin 4, Ireland

**Keywords:** neuromorphic computing, synaptic emulation, analog memristive device, electronic resistive switching, charge-trapping memristor, squaraine, nanowire

## Abstract

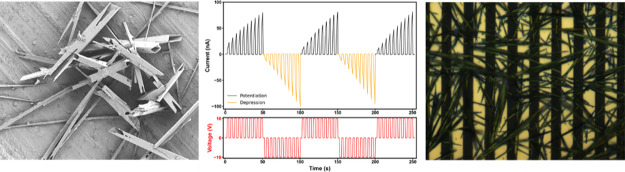

In this work, the discovery of volatile memristive devices that exhibit analog resistive switching (RS) and synaptic emulation based on squaraine materials is presented. Specifically, organic microtubes (MTs) based on 2,4-bis[(4-(*N*,*N*-diisobutyl)-2-6-hydroxyphenyl]squaraine (SQ) are prepared by evaporation-induced self-assembly (EISA). The MTs are ca. 2 μm in diameter (aspect ratio: 10–130). While powder X-ray diffraction data for MTs identify monoclinic and orthorhombic polymorphs, optical data report the monoclinic phase with energetic disorder. By favorable energetic alignment of the Au work function with the SQ HOMO energy, unipolar (hole-only) symmetric metal–insulator–metal devices are formed by EISA of MT meshes on interdigitated electrodes. The DC *I–V* characteristics acquired exhibit pinched hysteretic *I–V* loops, indicative of memristive behavior. Analysis indicates Ohmic transport at low bias with carrier extraction by thermionic emission. At high bias, space-charge-limited conduction in the presence of traps distributed in energy, enhanced by a Poole-Frenkel effect and with carrier extraction by Fowler-Nordheim tunneling, is observed. These data indicate purely electronic conduction. *I–V* hysteresis attenuates at smaller voltage windows, suggesting that carrier trapping/detrapping underpins the hysteresis. By applying triangular voltage waveforms, device conductance gradually increases sweep-on-sweep, with wait-time-erase or voltage-erase options. Using square waveforms, repeated erase-write-read of multiple distinct conductance states is achieved. Such analog RS behavior is consistent with trap filling/emptying effects. By waveform design, volatile conductance states may also be written so that successive conductance states exhibit identical current levels, indicating forgetting of previously written states and mimicking the forgetting curve. Finally, advanced synaptic functions, i.e., excitatory postsynaptic current, paired-pulse facilitation, pulse-dependent plasticity, and a transition from short- to long-term memory driven by post-tetanic potentiation, are demonstrated.

## 1 Introduction

The current approach to the emulation of neural behavior in artificial intelligence (AI) is based on algorithmic implementations on a software level, e.g., via deep neural networks, rather than emulation by hardware.^1^ Thus far, neural emulation has proven difficult because of the highly complex and interconnected nature of thought processes, i.e., parallelism. Traditional computing systems, based on a von Neumann architecture, suffer from high energy consumption and latency due to a huge amount of data transfer between the separated memory and logic units.^2^ To address these shortcomings, neuromorphic computing aims to implement artificial neural networks (ANNs) at the hardware level, with the goal of emulating the function and efficiency of the human brain on a compact chip, i.e., a hardware-based biomimetic brain.^3−5^ In this regard, analog memristors, two-terminal resistance switches with an inherent memory, can function as artificial synaptic elements in an ANN.^6,7^ Memristors bear a striking resemblance to biological synapses. The metal/insulator/metal (MIM) architecture often employed in these devices is analogous to the presynaptic neuron/synapse/postsynaptic neuron, respectively, in the brain. Also, the conductance of analog memristors can be incrementally modified by modulating the charge flux, akin to the evolution of synaptic weight between neurons, a process referred to as synaptic plasticity in neuroscience. In biology, this plasticity underlies the ability of the brain to compute, learn, and memorize.^8^

Recently, volatile memristors have been demonstrated, within which a voltage-programmed resistance state tends to undergo gradual relaxation toward a thermodynamically stable state following removal of the applied stimulus, offering the type of dynamics desirable for emulation of biological neurons and synapses.^9−11^ By exploiting this volatile behavior, short-term plasticity (STP), which lasts from seconds to tens of minutes before fading to its initial state, can be emulated by temporal enhancement of the device conductance. The conductance decay process affords these devices an “internal clock,” which can encode temporal information, emulating synaptic functionality such as spike-rate-dependent plasticity (SRDP). In this regard, Wang et al. demonstrated SRDP using a diffusive memristor, whereby the change in synaptic weight (conductance) was dependent on the time interval between voltage pulses, with shorter intervals inducing larger synaptic weight enhancements.^9^ Generally, this fading memory-type behavior resembles the “forgetting curve,” broadly developed since Ebbinghaus studied forgetting in 1885, and may enable the future implementation of artificial neural systems that emulate human memory. Further, in such devices, STP may be converted to long-term plasticity (LTP) through rehearsals, i.e., consolidation.^12,13^ Consequently, neuromorphic electronic synapses based on analog, volatile memristors are exciting candidates for realizing the goal of a hardware-based biomimetic brain.

By comparison, volatile memristors that exhibit binary or threshold resistive switching (RS) may be employed as neuronal elements in neural networks for neuromorphic computing, as their ability to exhibit dynamic resistance changes and respond to input spikes permits emulation of the neuron-like processing of information.^3^ Such memristors abide by the “all-or-nothing” rule observed in biological neurons; the threshold switching behavior enables the memristor to transition between distinct states in response to a certain level of input, resembling the characteristic binary firing observed in neurons. Consequently, volatile memristors with threshold switching may replicate this binary behavior, making them suited for emulating the spiking dynamics of artificial neurons in neuromorphic computing.

In this work, the discovery of volatile memristive devices that exhibit analog RS and synaptic emulation based on squaraine materials is presented. Squaraines are a class of small-molecule quadrupolar donor–acceptor–donor (D–A–D) chromophores that, in solution, exhibit sharp absorption bands along with intense fluorescence in the red and near-infrared.^14,15^ Intermolecular interactions between squaraines are known to give rise to the formation of molecular aggregates, accompanied by a significant modification of their electronic properties.^16^ In the condensed phase, squaraine polymorphs exhibit broad, even panchromatic absorption, along with likewise altered fluorescence.^17−19^ The broad solid-state absorption, coupled with a *p*-type semiconductor character, has found application in light harvesting devices, e.g., organic photovoltaics.^20−23^ Squaraine-based organic field-effect transistors have also been demonstrated.^24^ Squaraine-based nanomaterials have been reported, e.g., vapor-phase deposition of fibrillar structures and solution-based preparation of nanowires (NWs) for use in nanoscale photodetectors.^25−27^

**Scheme 1:**
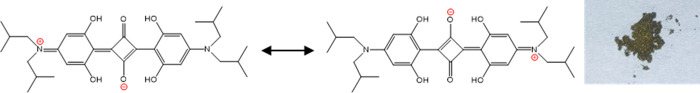
Left: Depiction of Two Resonant, Degenerate Zwitterionic (Betaine-Type) States of 2,4-Bis[(4-(*N*,*N*-diisobutyl)-2-6-hydroxyphenyl]squaraine (SQ), i.e., D^+^A^–^D and DA^–^D^+^; Right: SQ Powder Exhibits a Gold Color under Laboratory Light

Herein, an organic microtube (MT) mesh e-synapse based on the squaraine molecule 2,4-bis[(4-(*N*,*N*-diisobutyl)-2-6-hydroxyphenyl]squaraine (SQ) is reported; see Scheme 1. High-aspect-ratio SQ MTs are prepared by evaporation-induced self-assembly (EISA) from a solvent:nonsolvent mixture, and their structural and optical properties are characterized in detail. Carrier transport in MT meshes is studied and assigned to purely electronic conduction (Ohmic and space-charge-limited current (SCLC)) in the presence of traps. Also, *I–V* hysteresis, analog RS, and erase-write-read of multiple distinct conductance states are demonstrated and interpreted in terms of carrier trapping/detrapping effects. In addition, volatile conductance states are written with successive conductance states exhibiting identical current levels, indicating the forgetting of previously written states and mimicry of the forgetting curve. Finally, advanced synaptic functions, i.e., excitatory postsynaptic current (EPSC), paired-pulse facilitation (PPF), pulse-dependent plasticity, and a transition from short- to long-term memory driven by post-tetanic potentiation (PTP), are demonstrated. Operating by purely electronic RS, these novel organic semiconductor MT mesh devices provide a large dynamic range, access to multiple conductance states, linear and symmetric conductance tuning, and biorealistic synaptic emulation.

## 2 Experimental Section

### 2.1 Materials

All reagents and solvents were HPLC-grade and were used without further purification. The dye SQ (colored gold under laboratory light) was purchased from Sigma-Aldrich, Inc.; see Scheme 1. Deionized water (>16.1 MΩ cm) was used in the preparation of all aqueous solutions.

### 2.2 Single Crystal Preparation and Analysis

SQ crystals adopting the monoclinic phase were grown via the addition of methanol (40 mL) to a 1 mg mL^–1^ SQ/dichloromethane (DCM) solution (10 mL), followed by slow evaporation of the solvents. The X-ray intensity data were measured (λ = 0.71073 Å) at 100 K on a Bruker D8 Quest ECO instrument with an Oxford Cryostream low temperature device using a MiTeGen micromount. Bruker APEX software was used to correct for Lorentz and polarization effects. The structure was solved with the SHELXT structure solution program using intrinsic phasing and refined with the SHELXL refinement package using least squares minimization with Olex2. The single crystal structure data were visualized and analyzed with Mercury (2020.1 CSD Release), available free of charge from www.ccdc.cam.ac.uk/mercury/. Further details are available in the Supporting Information, Section SI.I.

### 2.3 Microtube Preparation

One mg of SQ powder was added to 1 mL of DCM solvent to yield a 1 mg mL^–1^ SQ/DCM solution. The vial was then sealed, and this solution was stirred on a hot plate for 1 h at 50 °C. After cooling, a 1 mL aliquot of the SQ/DCM solution was then added to a 3:2 water:ethanol solution (H_2_O:EtOH; 5 mL total volume) under vigorous stirring. After 3 min, stirring was ceased, and the mixture was allowed to stand for 1 h to permit phase separation to an aqueous layer (top) and an organic layer (bottom). To form MTs by EISA, a pipet was used to aspirate 20 μL of liquid from the organic layer and deposit it onto a solid substrate for drying in air (1 h).

### 2.4 Electrochemical and Spectroscopic Measurements

Cyclic voltammetry (CV) measurements were performed by using an EmStat Pico potentiostat (PalmSens BV). Experiments were carried out in DCM with 0.1 M TBA tetrafluoroborate as the supporting electrolyte at a dye concentration of 0.1 mM and a scan rate of 5 V s^–1^. Pt wires were used as both reference and counter electrodes, along with a boron-doped diamond working electrode. Ferrocene was used as the internal standard. UV–vis absorbance spectra were acquired by using a double-beam spectrophotometer equipped with a 60 mm integrating sphere (V-650; Jasco, Inc.). Photoluminescence (PL) spectra were acquired using a Quanta Master 40 (Photon Technology International, Inc.). X-ray photoelectron spectroscopy (XPS) was carried out with an Axis Ultra^DLD^ (Kratos, Ltd., UK) system using Al K_α_ radiation (1486.7 eV).

### 2.5 Structural Measurements

Optical microscopy image data was acquired in reflection mode using a calibrated upright epi-fluorescence microscope (BX51, Olympus Corp.) equipped with a 100 W halogen lamp, an X-Cite 120Q fluorescence lamp illuminator, and a thermoelectrically cooled color CCD camera. Scanning electron microscopy (SEM) data were acquired using a Zeiss Sigma 300 SEM equipped with a secondary electron detector. Powder X-ray diffraction (P-XRD) analysis was carried out on a D500 Kristalloflex diffractometer (SIEMENS) using Cu *K*_α_ radiation (λ = 0.154056 nm). Voltage and current were 40 kV and 30 mA, respectively, and each scan was conducted in 2θ/° mode, between 5 and 30°.

### 2.6 Electrical Measurements

Except where otherwise indicated, all measurements were carried out using interdigitated electrodes (IDEs) patterned on rigid glass substrates that comprised 125 interdigital electrode pairs (Au on glass; 250 fingers; thickness: 100 nm; length: 6.76 mm; width: 10 μm; gap width: 10 μm; G-IDEAU10, Metrohm Ireland, Ltd.). In one instance, flexible IDEs were employed for measurements. These comprised 10 interdigital electrode pairs (Cu(12 μm), Ni(3 μm), and Au(1 μm) layers on 60 μm poly(ethylene terephthalate) (PET); 20 fingers; length: 3.3 mm; width: 100 μm; and gap width: 100 μm). All measurements were carried out using a SP-200 potentiostat (BioLogic) in the dark. During *I–V* measurements, DC data were acquired in ultralow current mode at 200 mV s^–1^ unless otherwise stated. Data acquisition and analysis were undertaken using EC-Lab software.

## 3 Results and Discussion

### 3.1 Single Crystal Analysis

Single crystals of SQ were grown by preparing 10 mL of a 1 mg mL^–1^ SQ/DCM solution, followed by slow addition of 40 mL of methanol, allowing for two distinct layers to be observed (blue and clear, respectively). The solution was allowed to evaporate over several weeks, and clear green block-like crystals and metallic green rod-like crystals subsequently formed and were suitable for X-ray crystallography; see Figure S1. For SQ, two polymorphs are documented: a monoclinic phase (space group *P*2_1_/*c*) and an orthorhombic phase (space group *Pbcn*).^28,29^ Here, monoclinic and orthorhombic polymorphs were observed in the single crystal data.

**Figure 1:**
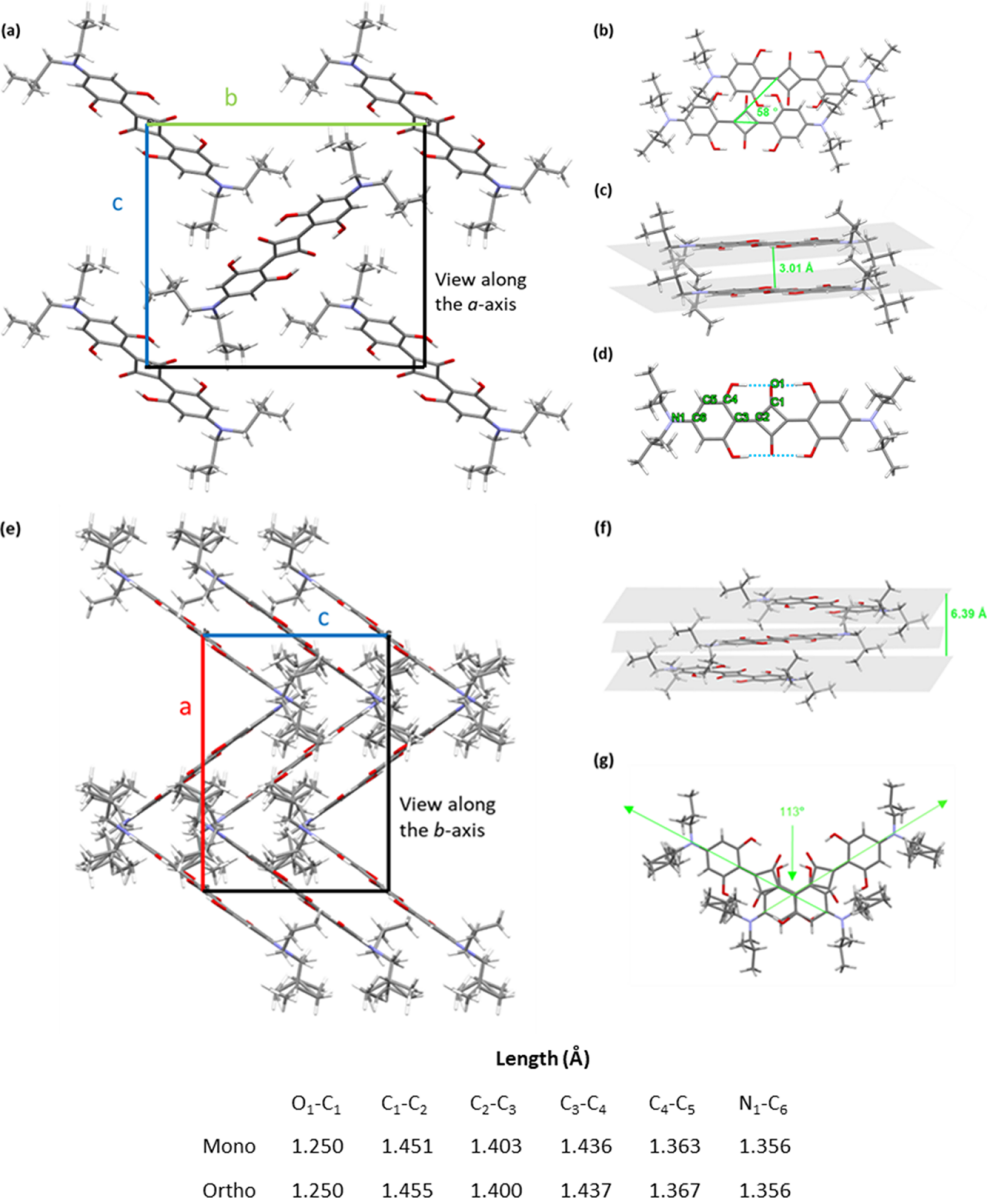
(a) SQ monoclinic polymorph exhibits classical herringbone packing within the crystallographic *bc*-plane and slipped π-stacking along the *a*-axis. Unit cell axes color coding: *b*: green, *c*: blue. (b) Nonprojected inclination angle θ of a dimer sandwich is 58°. (c) Interplanar distance of π-stacking is 3.01 Å. (d) Intramolecular H-bonding between hydroxyl groups and squaric oxygens is indicated by light blue lines. (e) The orthorhombic polymorph with herringbone packing can be understood as two interdigitating stacks that are rotated against each other. Unit cell axes color coding: *a*: red, *c*: blue. (f) The aromatic plane distance within a stack of parallel, aligned molecules is 6.39 Å. (g) The interdigitating stacks are rotated by 113° against each other (torsional angle). Selected bond lengths between labeled atoms are tabulated at the bottom.

The monoclinic polymorph adopts a classical herringbone packing (two SQ molecules per unit cell) within the crystallographic *bc*-plane and a slipped and inclined π-stacking along the *a*-axis; see Figure 1a. The nonprojected inclination angle of a dimer sandwich, θ, is determined to be 58°; see Figure 1b. All molecules within a stack show parallel and coplanar ordering. The aromatic interplanar distance is 3.01 Å; see Figure 1c. In each SQ molecule, the central core is stabilized by H-bonding, enabling the coplanar arrangement of the squaric and anilino rings; see Figure 1d, where H-bonding is indicated by blue dashed lines. Selected bond lengths between labeled atoms are also tabulated at the bottom of Figure 1. The O_1_–C_1_, C_2_–C_3_, C_4_–C_5_, and C_6_–N_1_ bonds appear to be shorter than a single bond (i.e., some double bond character), while, conversely, the C_1_–C_2_ and C_3_–C_4_ bonds exhibit some single bond character, suggesting the quinoidic structure of the anilino rings associated with the betaine-type arrangement; see Scheme 1, left.^18^ Unit cell parameters of the monoclinic SQ single crystal, with literature values for comparison, are summarized in Table 1.

**Table 1:** Unit Cell Parameters of Monoclinic and Orthorhombic SQ Crystal Samples

polymorph	*a* (Å)	*b* (Å)	*c* (Å)	β (°)	*Z*
*P*2_1_/*c* (this work)	6.1893(8)	16.4482(19)	14.3989(18)	92.351(5)	2
*P*2_1_/*c* (ref (28))	6.2034(16)	16.478(4)	14.518(4)	92.406(4)	2
*Pbcn* (this work)	15.0597(7)	18.1899(9)	10.7693(5)	90	4
*Pbcn* (ref (29))	15.0473(8)	18.1959(10)	10.7775(6)	90	4

The orthorhombic polymorph also adopts a herringbone packing (four SQ molecules per unit cell); see Figure 1e. The aromatic plane distance within a stack of parallel, aligned molecules is 6.39 Å; see Figure 1f. This packing can be understood as two interdigitating stacks rotated by 113° against each other; see Figure 1g. The neighboring molecules within a stack are rotated in an alternating manner. The aromatic planes of two stacked molecules are not parallel but rather are tilted by 4.44°. From the tabulated bond lengths at the bottom of Figure 1, the quinoidic structure of the anilino rings associated with the betaine-type arrangement is again suggested; see Scheme 1, left. Unit cell parameters of the orthorhombic SQ single crystal, with literature values for comparison, are summarized in Table 1.

### 3.2 Microtube Preparation and Characterization

Previously, a methyl-substituted anilino squaraine was shown to undergo self-assembly following addition of a squaraine/DCM solution (good solvent) to a 1:1 (v/v) H_2_O:EtOH (poor solvent) with subsequent deposition onto a substrate; during air drying, NW formation occurred with squaraine aggregation in the slipped-stack arrangement.^26^ This is in accordance with the accepted picture of the squaraine ground and excited states as being donor–acceptor–donor intramolecular charge-transfer states and molecular aggregation in the slipped-stack arrangement being directed by the strong intermolecular interactions that arise between the donor and acceptor groups.^18^ Squaraine aggregation and growth into NWs in the slipped-stack arrangement is thus likely directed both by strong intermolecular interactions and by a reduced solubility of the molecule in the mixed-solvent system.^26^

**Figure 2:**
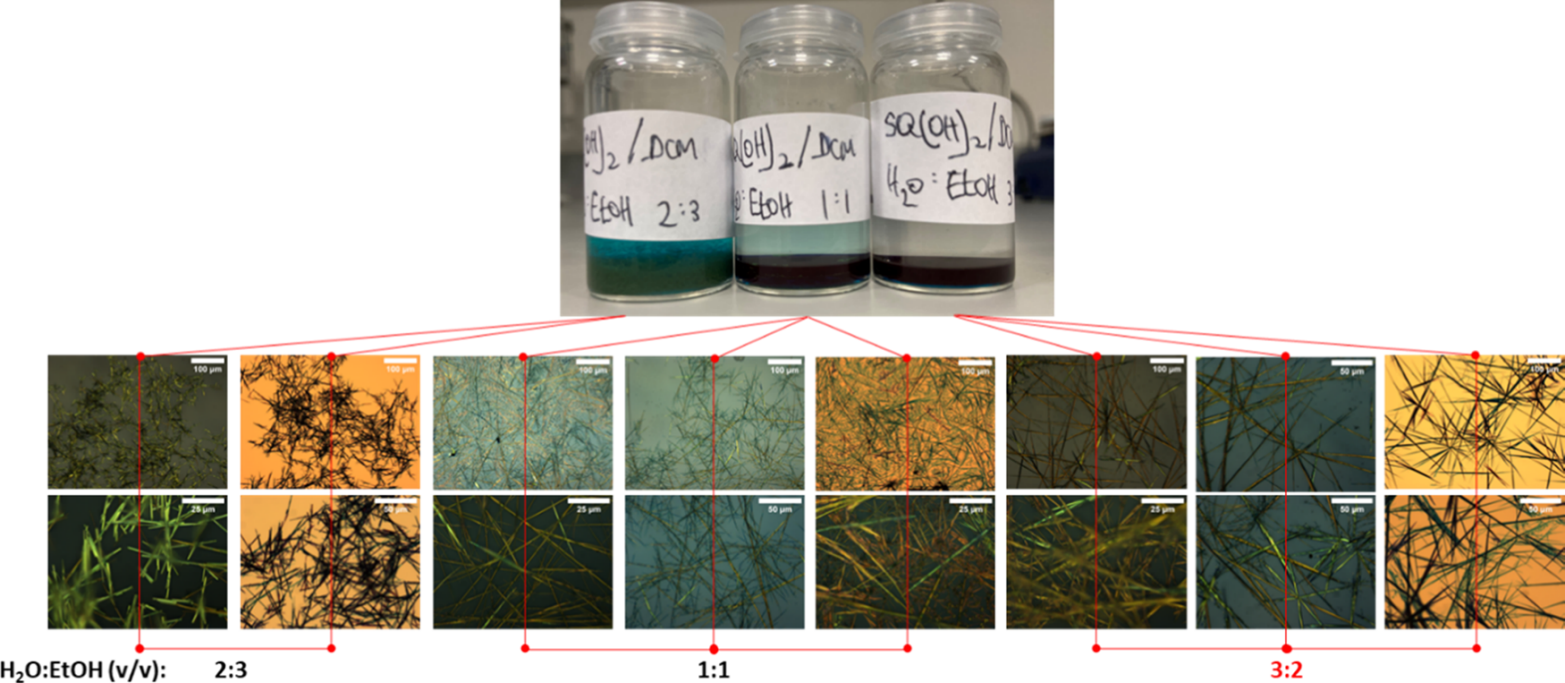
(Top) Photographs of sample vials used for nonsolvent (H_2_O:EtOH) screening in high-aspect-ratio fiber formation and (Bottom) corresponding reflected light optical microscopy images of the resulting materials; each H_2_O:EtOH ratio (v/v) is indicated.

Here, this approach was refined for the SQ molecule by screening a range of formulations. Specifically, a 1 mL aliquot of a 1 mg mL^–1^ SQ/DCM solution was added to a series of 5 mL of H_2_O:EtOH solutions (2:3, 1:1, and 3:2 (v/v)) under vigorous stirring. After 3 min, stirring was ceased, and the mixture was allowed to stand for 1 h to permit phase separation to an aqueous layer (top) and an organic layer (bottom). Photographs of each vial (1 h) are shown in Figure 2, top. For samples with 1:1 and 3:2 H_2_O:EtOH, aliquots from the organic layer were transferred from each preparation vial onto appropriate inspection substrates via pipet aspiration where precipitation of solid SQ material subsequently occurred during drying. For the 2:3 H_2_O:EtOH sample, no distinct phase separation occurred, and a green-colored precipitate was observed. Consequently, a sample of precipitated solid material plus liquid was transferred from the preparation vial onto an appropriate inspection substrate via pipet aspiration. Optical microscopy images of all samples are shown in Figure 2, bottom. For 2:3 H_2_O:EtOH, rod-like morphologies were observed in the precipitate, whereas for 1:1 and 3:2 H_2_O:EtOH, higher aspect ratio SQ morphologies with amorphous SQ material were apparent. Importantly, the 3:2 H_2_O:EtOH sample contained abundant SQ fibers, likely formed successfully by in situ EISA with a low background of amorphous material.^26^

**Figure 3:**
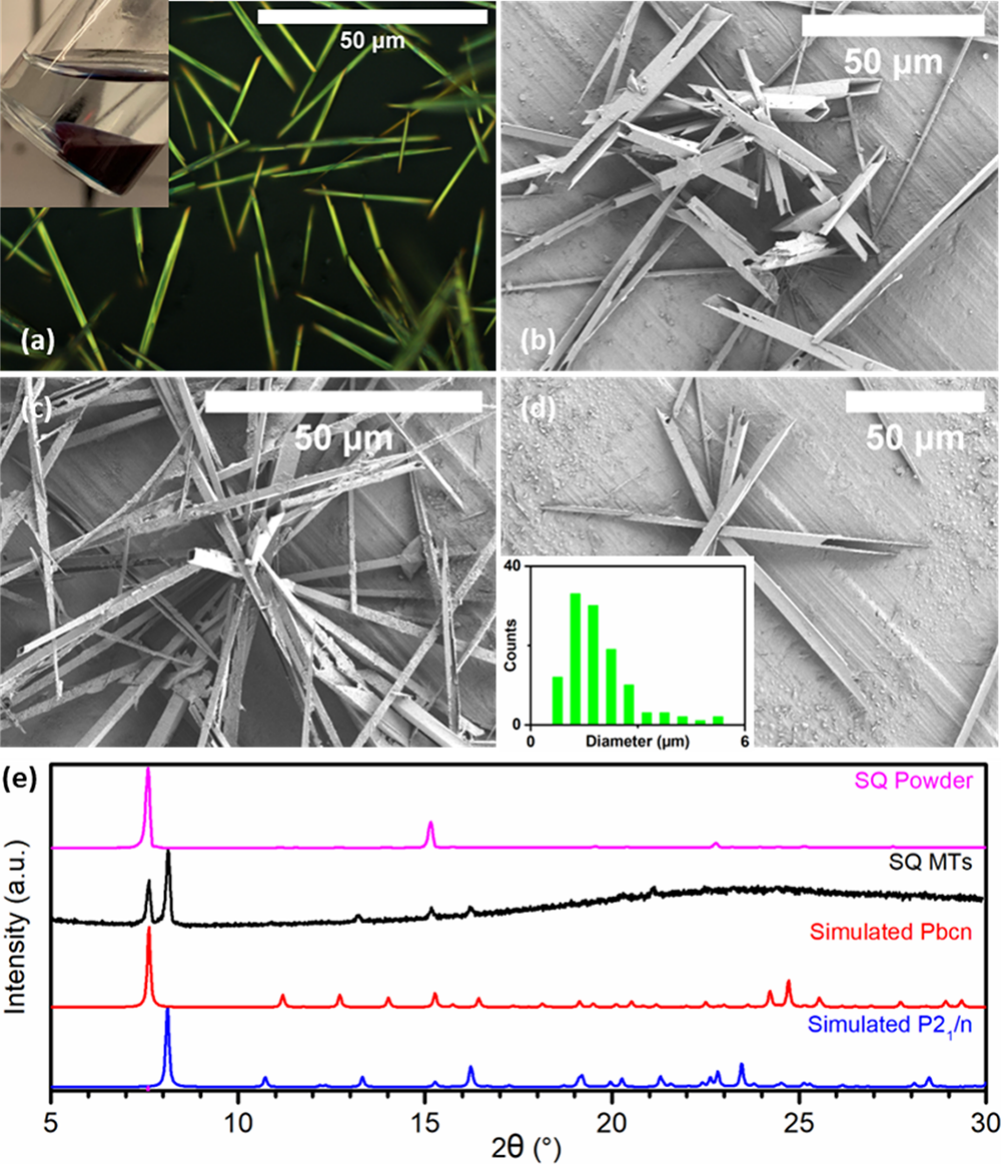
(a) Reflected light optical microscopy image of SQ MTs prepared using 3:2 H_2_O:EtOH (v/v); inset: sample preparation vial with visible separation of the phases. (b–d) SEM images of the MTs; inset in (d): size histogram of MT diameters. (e) P-XRD data with simulated diffractograms of the SQ polymorphs.

Further imaging of a 3:2 H_2_O:EtOH SQ sample by reflected light optical microscopy confirmed that this material comprised a dense mesh of randomly distributed one-dimensional SQ fibers with a gold color; see Figure 3a. The microtubular (MT) morphology of the fibers was then revealed by SEM (Al foil substrate); see Figure 3b–d. Analysis of SEM image data gave an average SQ MT diameter of ca. 1.9 ± 0.9 μm (with aspect ratios of 10–130); see Figure 3d, inset. Interestingly, SEM images of fibers prepared using 0.1 mg mL^–1^ SQ/DCM solutions are shown in Figure S3. The square profile of the structures is apparent. At lower [SQ], the structures were smaller; analysis of image data gave an average diameter of ca. 0.8 ± 0.2 μm. Returning to the 3:2 H_2_O:EtOH SQ sample being considered here, XRD data acquired for SQ MT mesh samples indicated the presence of both monoclinic *P*2_1_/*c* and orthorhombic *Pbcn* packing arrangements by comparison with simulated diffractograms of the two known SQ polymorphs (Cambridge Crystallographic Data Centre); see Figure 3e.^28,29^ Benchmark data acquired for the as-received SQ powder indicated orthorhombic Pbcn packing only.

**Figure 4:**
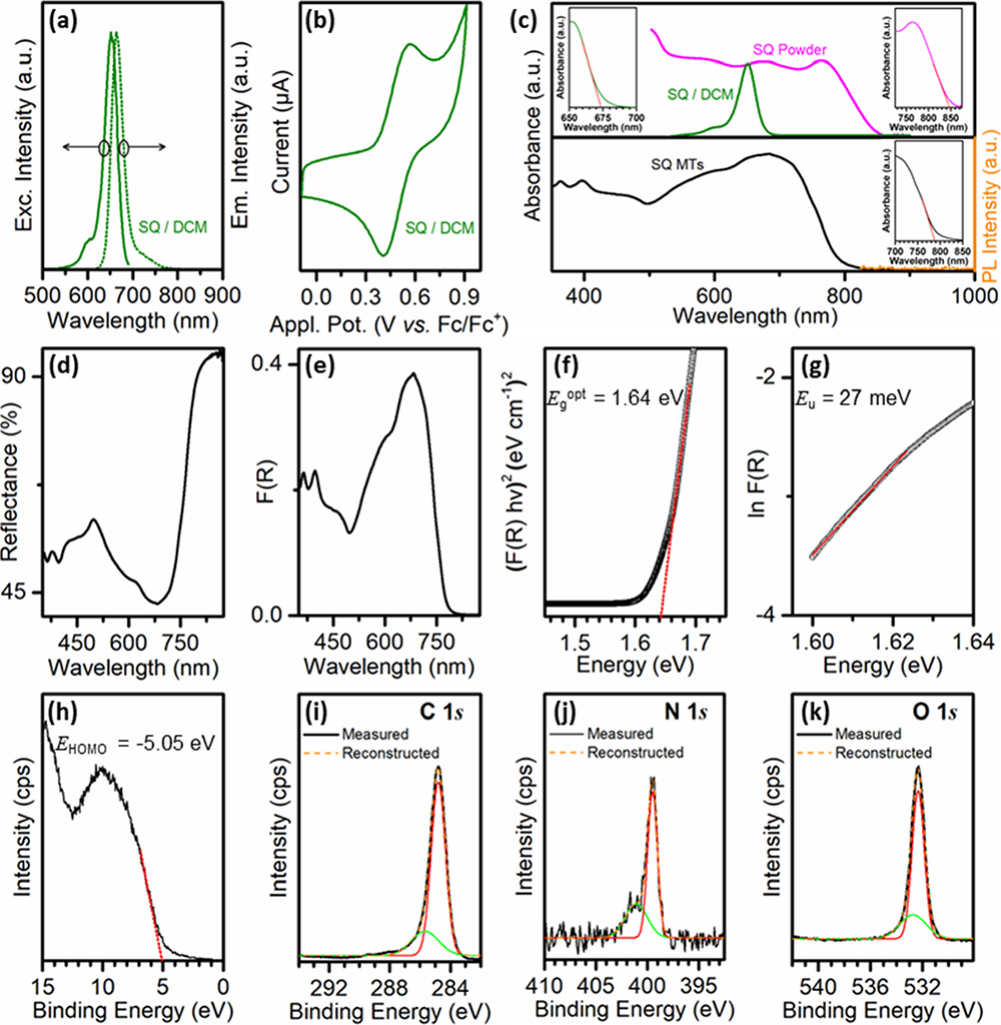
(a) PL excitation and emission spectra for SQ/DCM. (b) Cyclic voltammetry for SQ/DCM. (c) UV–vis absorbance of (Top) SQ/DCM and SQ powder and (Bottom) a SQ MT mesh, with PL emission; insets: absorbance onsets by linear extrapolation. (d) Diffuse reflectance for SQ MT mesh. (e) Kubelka–Munk absorption curve. (f) Tauc plot. (g) Urbach energy plot. High resolution XPS data for MTs in (h) the valence band region and (i–k) the C 1*s,* N 1*s,* and O 1*s* bands.

Primary spectroscopy and electrochemical data for a SQ/DCM solution were acquired. PL excitation (λ_em_ = 700 nm) and emission (λ_exc_ = 610 nm) spectra are shown in Figure 4a. An excitation maximum at 652 nm was observed, while the emission maximum occurred at 664 nm (Stokes shift of 12 nm). Using CV data for SQ/DCM, a value for the one-electron oxidation potential (*E*_ox_) of 0.51 V (vs Fc/Fc^+^) was estimated, yielding a value of −5.3 eV for the HOMO energy level, *E*_HOMO_ (monomer); see Figure 4b. UV–vis absorbance data for SQ/DCM were also acquired; see Figure 4c, top. An absorbance maximum at 652 nm was observed while a shoulder in the absorbance spectrum, characteristic of vibrational fine structure, was also apparent around 600 nm.^19^ The optical energy gap, *E*_g_^opt^ (monomer), was estimated by linear extrapolation of the absorption data to the *x*-axis near the onset of optical absorbance in the low energy region, yielding a value of 672 nm or 1.84 eV; see Figure 4c (inset), top. Combining this with the value for *E*_HOMO_ of −5.3 eV gave a value of −3.46 eV for the LUMO energy level, *E*_LUMO_ (monomer). The values for *E*_HOMO_ (monomer), absorbance maximum, *E*_g_^opt^ (monomer), and *E*_LUMO_ (monomer) agree with literature reports.^22,30^

Solid-state UV–vis absorbance data were acquired for the as-received SQ powder and SQ MT mesh samples; see Figure 4c, top and bottom, respectively. These spectra were both significantly different from the solution spectrum of the monomeric SQ species being broadened and practically panchromatic, with light absorption across much of the visible wavelength range. Also, PL emission was not detectable for the SQ MT mesh; see Figure 4c, bottom. Such changes in optical properties following aggregation into the solid state are characteristic of various anilino-squaraine compounds.^19^ Further, the onset of absorbance of the SQ powder sample (∼850 nm) was significantly red-shifted with respect to that of the MT mesh sample (∼790 nm); see Figure 4c, top and bottom insets, respectively. In this regard, previous studies have reported a > 800 nm onset of optical absorbance for orthorhombic SQ and a < 800 nm onset for the monoclinic polymorph.^18,31^ Taken together, the optical properties of the SQ powder (orthorhombic) and MT mesh samples (orthorhombic and monoclinic) suggest that the MT samples were dominated by the monoclinic phase, as the lower energy onset of absorbance observed for the SQ powder did not manifest at the corresponding energy in the MT sample.

To analyze the optical absorption properties of the SQ MTs in more detail, the UV–vis diffuse reflectance spectrum measured for a SQ MT mesh sample is shown in Figure 4d. The data were transformed using the Kubelka–Munk function, *F*(*R*) = *K*/*S* = (1 – *R*)^2^/(2*R*), where *K* is the absorption coefficient, *S* is the scattering coefficient, *R* (%) is the reflectance of the sample, and *F*(*R*) is the Kubelka–Munk function, i.e., absorption; see Figure 4e.^32^ To estimate the optical energy gap of a semiconductor, *E*_g_^opt^, the energy-dependent absorption coefficient, α, may be expressed as (α*h*ν)^*n*^ = *B*(*h*ν – *E*_g_^opt^), where *h*ν is the photon energy, *B* is a constant, and *n* indicates the nature of the transition, e.g., *n* = 1/2 for an indirect transition and *n* = 2 for a direct transition.^33,34^ As the absorption coefficient, α, is proportional to *F*(*R*), *F*(*R*) may substitute for α yielding [*F*(*R*)*h*ν]^*n*^ = *B*(*h*ν – *E*_g_^opt^). Consequently, plotting [*F*(*R*)*h*ν]^2^ against *h*ν yielded the Tauc plot for this direct bandgap semiconductor and an estimated value for *E*_g_^opt^ of 1.64 eV by linear extrapolation of the data to the *x*-axis; see Figure 4f.

Also, an Urbach energy tail is apparent in the absorbance spectrum, which, in structurally disordered semiconductors, may be assigned to optical transitions between localized (intragap) states and extended states; see Figure 4e.^35^ This suggests the presence of an energetic disorder in the MTs. The rapid room-temperature conditions of EISA may cause the formation of localized trap states, e.g., structural defects/disorder and polycrystallinity, in the SQ MTs. Defect states or traps may also arise due to environmental effects, e.g., effects of temperature, moisture, ambient gases (O_2_), and light exposure.^36^ To estimate the extent of energetic disorder (Urbach energy, *E*_u_), ln (*F*(*R*)) vs *E* was plotted at low energy, where α ∝ *F*(*R*), and the data were fitted by α = α_0_ exp (*E/E*_u_), where α is the energy-dependent absorption coefficient, α_0_ is a constant, and *E = h*ν, to estimate an *E*_u_ of 27 meV; see Figure 4g.^37^

XPS measurements in the valence band region were made on a SQ MT mesh sample, and a value of −5.05 eV for the HOMO energy, *E*_HOMO_, was estimated by linear extrapolation; see Figure 4h. By addition of *E*_g_^opt^ (1.64 eV) to *E*_HOMO_, a LUMO energy, *E*_LUMO_, of −3.41 eV was obtained. Data acquired at higher energies gave core-level spectra for C 1*s*, N 1*s*, and O 1*s* that were deconvoluted by iterative fitting and reconstruction to investigate bonding environments; see Figure 4i–k. Binding energies were referenced to the center of the broad C 1*s* peak (284.8 eV), placing the binding energy of the main N 1*s* peak at 399.5 eV in agreement with values reported for related aminophenyl-substituted squaraines.^38^ The C 1*s* band was decomposed into two peaks centered at 284.8 eV (C in the four- and six-membered rings) and 285.7 eV (C–O and C=N).^39^ The N 1*s* band was deconvoluted to two peaks centered at 399.5 eV (C=N) with a moderately intense satellite at 401.1 eV (shakeup processes).^40^ The O 1*s* band deconvolution exhibited peaks at 532.3 eV (C–O) and 532.7 eV (“excess” oxygen from adsorbed O_2_ and/or H_2_O).^39,40^ Overall, these assignments were consistent with the quinoidic structure of the anilino rings associated with the betaine-type arrangement indicated by the single crystal XRD data; see Scheme 1, left.

### 3.3 DC Electrical Measurements

**Figure 5:**
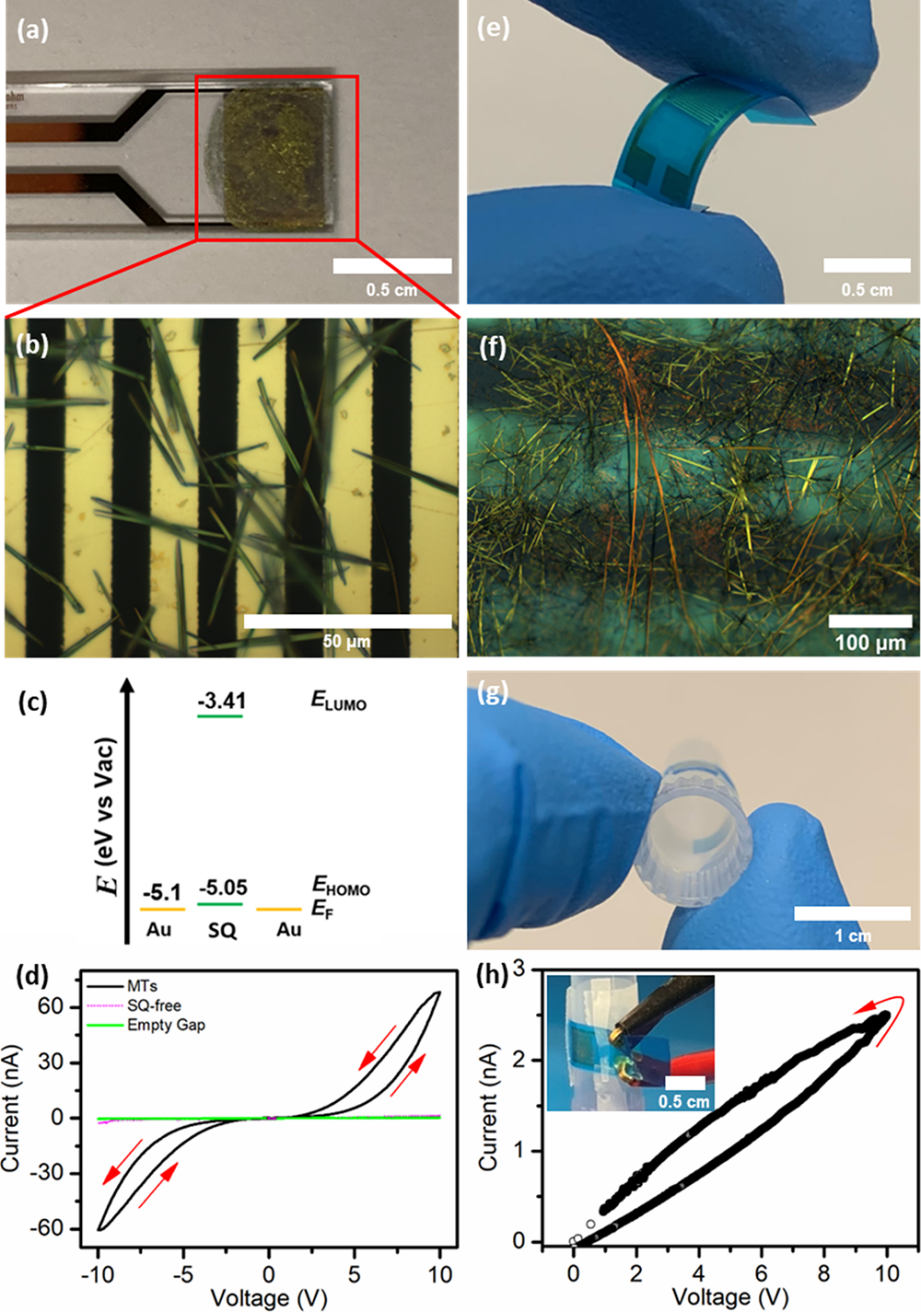
(a, b) Photograph and reflected light optical microscopy image, respectively, of SQ MT mesh on IDE array on glass. (c) Au-MTs-Au energy level diagram. (d) *I–V* loops acquired for MT mesh, with controls, on a linear scale. (e) Photograph of Au IDEs on PET. (f) Reflected light optical microscopy image of SQ MTs on the IDE array (a Au electrode is vertically orientated in the center of this image). (g) Photograph of IDEs attached to a standard laboratory 1 mL pipet tip (bend radius of 4 mm). (h) *I–V* loop acquired for the MT mesh device under bending (inset photograph).

SQ MT mesh devices were prepared in a lateral, symmetric MIM configuration by in situ EISA on Au IDEs on glass; see Figure 5a. The MTs were of comparable dimensions (average diameter of ca. 1.7 ± 0.9 μm) to those formed on Al foil with many bridging MTs formed atop the Au IDEs, as indicated by reflected light optical microscopy; see Figure 5b. An energy level diagram for the Au-MT mesh-Au device was drafted using the values of ca. −5.05 eV for *E*_HOMO_, 1.64 eV for *E*_g_^opt^, and −3.41 eV for *E*_LUMO_ determined above for the MTs; see Figure 5c. Given the favorable energetic alignment between the work function of Au (ca. −5.1 eV) and the HOMO energy of SQ, Au was considered an appropriate electrode material.

The DC *I–V* characteristics acquired from a typical MT mesh device were plotted in a linear format; see Figure 5d. A positive voltage sweep (0 V → +10 V → 0 V) was performed, followed by an analogous sweep in the reverse bias direction (0 V → −10 V → 0 V). The device displayed pinched hysteretic *I–V* loops, indicative of memristive behavior.^41^ As a *p*-type semiconductor with a LUMO energy considerably higher than the work function of the gold contact electrodes, hole-only transport through the SQ MT mesh was considered likely.^26^ The comparative symmetry of the *I–V* loops acquired during scans to positive and negative bias indicated negligible, if any, rectification, consistent with the symmetrical device configuration. Also, *I–V* characteristics measured for an empty IDE (i.e., empty gap) and for an IDE treated only with the solvent:nonsolvent combination (i.e., SQ-free) both exhibited ca. 1 nA currents without hysteresis, consistent with negligible contribution of leakage or background currents to the MT mesh response; see Figure 5d.

To investigate the use of alternative electrode substrates, SQ MT meshes were prepared by EISA on Au IDEs on a flexible PET substrate in a lateral MIM-type device configuration; see Figure 5e,f. The IDEs were attached to a standard laboratory 1 mL pipet tip to give a bend radius of 4 mm and electrically probed; see Figure 5g,h, inset. A positive voltage sweep (0 V → +10 V → 0 V) was applied. The measured current was ∼3 nA at +10 V, understandably lower than that of standard IDE/glass devices due to fewer electrode pairs with larger interelectrode spacing providing fewer bridging MTs; see Figure 5h. Importantly, the device displayed the pinched hysteretic *I–V* loop while bent, highlighting the feasibility of future flexible SQ MT mesh-based memristive devices.

**Figure 6:**
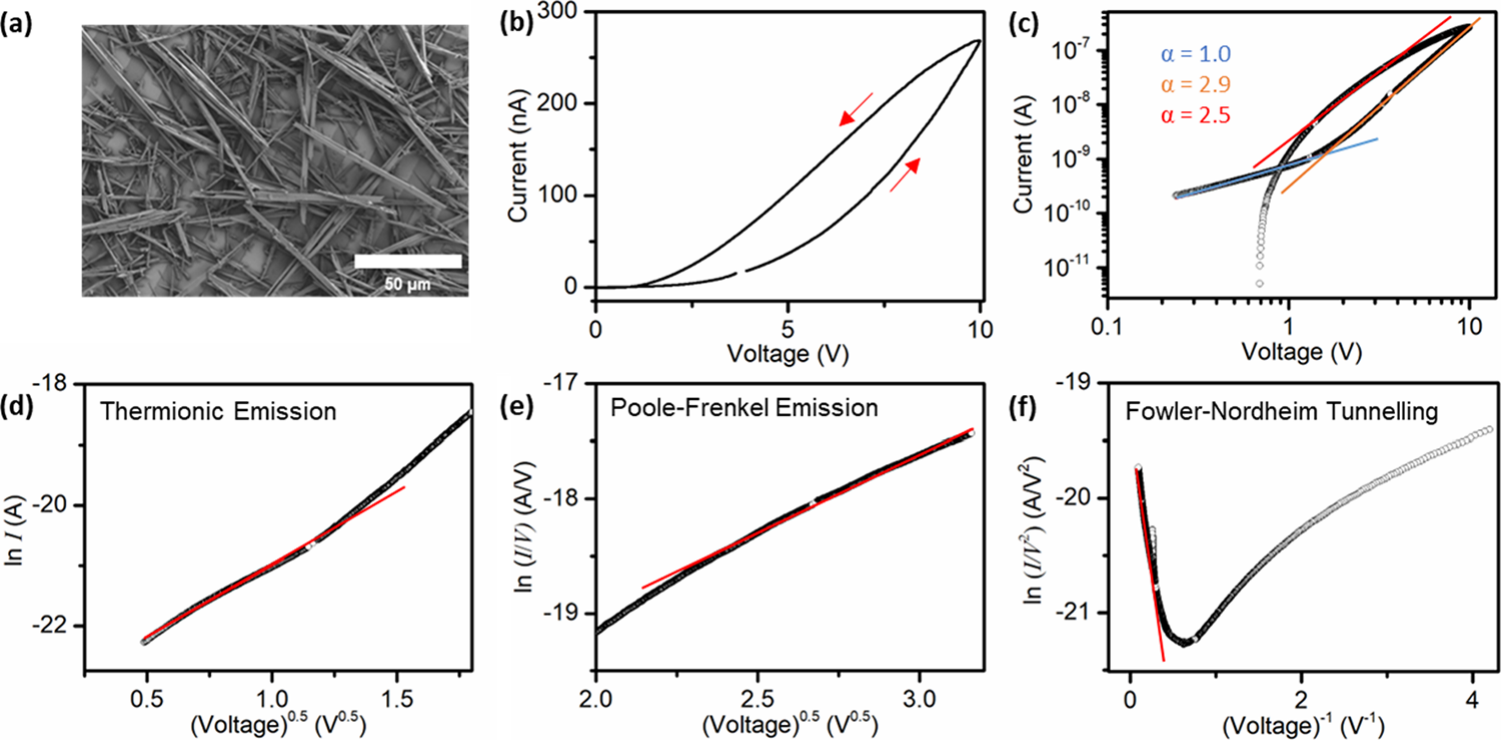
(a) SEM image of SQ MT mesh on IDE array. (b) Positive bias *I–V* loop acquired for MT mesh on a linear scale. (c) Positive bias *I–V* loop on a double logarithmic scale; slopes (α) of linear regions obtained by linear regression fits (*R*^2^ > 0.99). (d) TE fit at low bias. (e) PF fit at high bias. (f) FN fit at high bias.

To further characterize the transport behavior, a MT mesh device was prepared by double depositions on IDEs on glass; see Figure 6a. A positive voltage sweep (0 V → +10 V → 0 V) was performed, and the DC *I–V* characteristics acquired from the device were plotted in a linear format (Figure 6b). The device displayed a hysteretic *I–V* loop. A double logarithmic plot of the data is shown in Figure 6c. On the outward excursion (0 → +10 V) and between 0 V and +1.6 V (‘low bias region’), the data were fit by a line of slope α = 1.0, indicative of Ohmic transport, as described by
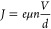
1where *J* is the current density, *e* is the electronic charge, μ is the hole drift mobility, *n* is the thermally generated carrier (hole) concentration, *V* is the applied voltage, and *d* is the sample thickness; see Figure 6c, blue line.^42^ In the Ohmic regime, the density of injected carriers is much less than the density of thermally generated intrinsic charge carriers, leading to a linear behavior with *I* ∝ *V*^α^ (α = 1). Concerning charge injection/extraction, when plotted as ln (*I*) versus *V*^0.5^, the data of the outward excursion exhibited a linear behavior in the low bias region; see Figure 6d. This observation suggested thermionic emission (TE), where
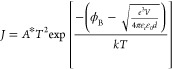
2and A* = 4π*ek*^2^m*/ h^3^ = 120m*/m_0_ is the effective Richardson constant, *m*_0_ is the free electron mass, m* is the effective electron mass, *T* is absolute temperature, ϕ_B_ is the Schottky barrier height, *k* is Boltzmann’s constant, *h* is Planck’s constant, ε_0_ is the vacuum permittivity, and ε_r_ is the relative permittivity of the semiconductor.^42^ The observation of TE may indicate that, while there was likely no barrier to hole injection at low bias, a small barrier to hole extraction was present.

On the outward excursion at applied biases above +1.6 V (‘high bias region’), the *I–V* data were fit by a line of slope α = 2.9; see Figure 6c, orange line. This power law relationship with *I* ∝ *V*^α^ (α > 2) is indicative of carrier transport by SCLC in the presence of traps that are exponentially distributed in energy.^3,36,42−44^ Generally, SCLC arises when an injecting electrode is in Ohmic contact (no or small injection barrier) with an organic material. At a sufficiently high voltage, the concentration of injected carriers exceeds that of thermally generated carriers. As the electrode has effectively injected more charge carriers than can be readily transported, a space charge region accumulates, which affects the current flow.

Charge trapping is an almost ubiquitous phenomenon in organic materials, and trap effects have been reported for squaraine solids (bulk heterojunction photodetector), with models for dark-injection SCLC being required to incorporate specific trap state distributions in hopping transport descriptions.^45,46^ In the presence of traps, charge transport by SCLC is influenced by the trapping and detrapping of carriers; a certain fraction of injected charge carriers will not participate in transport due to being captured by traps, resulting in a decrease in current compared with a trap-free system, denoted as trap-limited SCLC (TL-SCLC).^47^

In this regard, it has been shown that SCLC transport in the presence of traps exponentially distributed in energy above the HOMO level yields an *I*–*V* relationship of the type
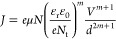
3where *N* is the effective density of transport states at the HOMO level, *N*_t_ is the effective density of trap states with respect to the HOMO level, and *m* (*m* = α – 1) is the quotient *T*_t_*/T*, where *T*_t_ (>*T*) is the temperature characterizing the exponential distribution of traps.^36,43,44^ This relationship describes Ohmic current flow when *m* = 0 (α = 1), trap-free SCLC (TF-SCLC) when *m* = 1 (α = 2), and TL-SCLC in the presence of traps when *m* > 1 (α > 2).^3,42^ As noted, a value for the Urbach energy, *E*_u_ = 27 meV, in the SQ MTs was extracted from optical data to quantify the extent of energetic disorder associated with shallow intragap trap states. Using *E*_u_ as a proxy for trap activation energy, *E*_A_ = *kT*_t_, gives *T*_t_ = 313 K, and α = 2.1 at *T* = 293 K, in approximate agreement with α = 2.9. Subsequently, when the outward excursion was terminated at +10 V, the device apparently remained in the TL-SCLC regime (*I* ∝ *V*^α^, α > 2), i.e., trap filling was incomplete.

Interestingly, when plotted as ln (*I/V*) versus *V*^0.5^, the *I–V* data exhibited a linear behavior at high bias indicative of Poole-Frenkel emission as described by
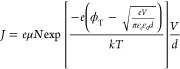
4where ϕ_T_ is the potential barrier associated with a trap; see Figure 6e.^48^ This observation suggests that the TL-SCLC conduction at high bias was enhanced by the Frenkel effect (reduction of effective trap depth at high electric fields). Concerning charge injection/extraction at high bias, when plotted as ln (*I/V*^2^) versus *V*^–1^, the data exhibited a linear dependence with a negative slope, indicative of Fowler-Nordheim (FN) tunneling; see Figure 6f. The *I*–*V* relationship may be expressed as
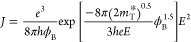
5where *m*_T_^*^ is the tunneling effective mass in the insulator and *E* is the electric field.^42^ This observation suggests that, while there was likely no barrier to hole injection at high bias, hole extraction occurred via FN tunneling. Additionally, TE current is expected to manifest as a curve of positive slope in a FN plot, and this behavior was apparent in the data.

On the return excursion (+10 → 0 V), a hysteresis was observed in the current flow where the current was enhanced compared with the outward excursion, likely due to the involvement of traps. In this regard, on the initial outward excursion described above, an exponential distribution of traps was proposed to become energetically accessible beyond +1.6 V, facilitating the trapping of a proportion of injected carriers and yielding TL-SCLC transport. On the return excursion, trapped carriers are expected to be gradually released, contributing to differential current. As a result, proportionately more carriers are expected to become available, enhancing the current and causing hysteresis to become apparent.^36^ Hole trapping-induced hysteresis has been observed in two-terminal organic devices, where SCLCs were affected by the trapping/detrapping of carriers.^49,50^ Also, the current in the intermediate region of the return sweep (+4.5 to +1.1 V) exhibited the TL-SCLC character (α = 2.5) of the high bias region of the outward excursion (α = 2.9). Finally, when the applied bias reached 0 V on the return excursion, the two branches of the *I–V* loop passed through the origin. We note that similar phenomena were observed during DC electrical measurements of devices under illumination; see Supporting Information, Section SI.III.

**Figure 7:**
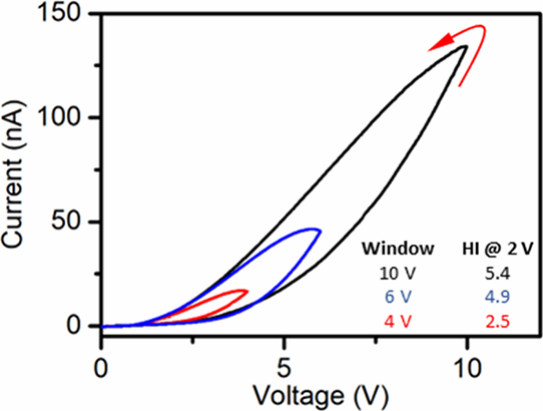
Plot of positive bias *I–V* loops acquired for an MT mesh using different voltage windows.

To further investigate the proposed role of carrier detrapping in contributing to enhanced current flow on the return excursion (*I–V* hysteresis), a positive voltage sweep was applied to a device in the dark for various voltage windows, and hysteretic index (HI) values were estimated; see Figure 7. The HI may be described by (*I*_on_ – *I*_off_)/*I*_off_, where *I*_on_ and *I*_off_ are the current magnitudes recorded at 2 V in the low resistance state (LRS; return excursion) and the high resistance state (HRS; outward excursion), respectively. The HI values decreased with decreasing voltage window employed as 5.4 (10 V), 4.9 (6 V), and 2.5 (4 V) suggesting that the contribution of charge trapping/detrapping to differential current becomes more pronounced with increasing applied bias window.^51^

### 3.4 Memristive Device Behavior

**Figure 8:**
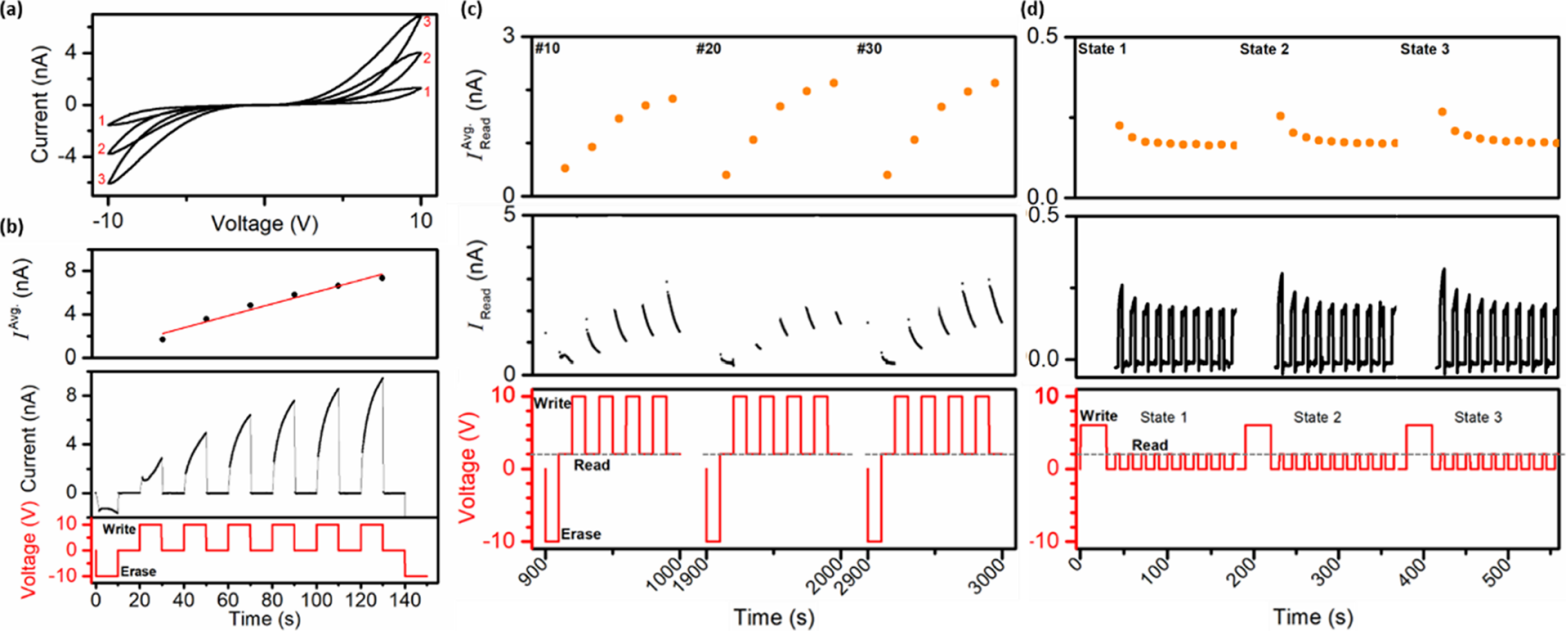
(a) Current–voltage trace obtained for a SQ MT mesh device during application of a triangular voltage waveform: three successive positive sweeps (0 V → +10 V → 0 V) followed by three successive negative sweeps (0.2 V s^–1^). Current–time traces obtained during application of a square voltage waveform: (b) a −10 V erase pulse followed by six consecutive +10 V write pulses with interleaved 0 V pulses and a final erase (all pulses 10 s); (c) forty cycles consisting of a −10 V erase pulse followed by a +2 V read pulse and four consecutive +10 V write pulses with interleaved +2 V read pulses (all pulses 10 s); and (d) a write pulse of +6 V (30 s) followed by ten consecutive read pulses of +2 V (5 s) interleaved with 0 V (10 s) pulses.

To explore the memristive behavior, voltage sweeps were applied to a device as follows: three successive positive sweeps (0 V → +10 V → 0 V) were performed, followed by three successive sweeps in the reverse bias direction (triangular waveform; scan rate: 0.2 V s^–1^); see Figure 8a. The conductance of the device gradually increased in an analog manner from one sweep to the next.^3,52,53^ As proposed above, this memory effect is consistent with a simple model of carrier injection, trapping/detrapping within a distribution of trap states, and extraction, as follows: during the first voltage sweep, trapping of injected carriers on the outward voltage excursion yields high bias transport in the TL-SCLC regime and a current level that characterizes the first conductance state. On the return excursion, trapped carriers are gradually released, contributing to a differential current that manifests in the *I*–*V* data as a hysteresis. However, as some trapped carriers likely persist in trap states in the material after the first sweep (i.e., on return to 0 V), these occupied traps are rendered inactive on the outward excursion of the second voltage sweep. Consequently, proportionately more injected carriers become available, and the current is enhanced, characterizing the second conductance state. On the second return excursion, the current due to free and detrapped carriers again causes *I*–*V* hysteresis. After this second sweep (on return to 0 V), an increased number of carriers likely remain trapped, comparatively more (occupied) traps are rendered inactive on the outward excursion of the third voltage sweep, and a further current enhancement characterizes the third conductance state. Eventually, over successive sweeps, progressive trap filling will cause the device conductance to approach a maximum value, and differences in current flow between successive conductance states will gradually decrease.^54,55^

To further illustrate the ability to tune the device conductance, a square voltage waveform was employed; see Figure 8b. Following an erase pulse of −10 V (to empty traps), six write pulses of +10 V were applied, with interleaved 0 V pulses, followed by a final erase pulse (all pulses 10 s). The six consecutive positive bias voltage pulses established six conductance states, and the average current value for each state, *I*^Avg.^, was plotted. The increase in *I*^Avg.^ from State 1 → State 6 was fitted by linear regression (*R*^2^ value: 0.96), the linearity of response being likely due to enhanced carrier detrapping over 10 s at 0 V. The ability to incrementally increase device conductance, analogous to the strengthening of synaptic weights between neurons in the brain during learning, indicates that the SQ MT mesh devices exhibit synaptic emulation.^56^

To demonstrate write/read, conductance states were created using a write voltage, and then probed using a read voltage (to minimize read disturbance, the read voltage, *V*_rd_, was smaller than the write voltage, *V*_rd_ = 0.2 × *V*_wr_).^57^ Specifically, each voltage cycle applied to the device consisted of an erase pulse of −10 V (10 s) and a read pulse of +2 V (10 s), followed by four consecutive write pulses of +10 V (10 s) with interleaved read pulses of +2 V (10 s); see Figure 8c, red trace. On completion of the first cycle, the process was repeated for 40 cycles (4000 s). The read currents specifically recorded during cycles 10, 20, and 30 are displayed as black traces, and each of the device conductance states is identified, e.g., HRS (State 1), MRS (medium resistance state; States 2–4) and LRS (State 5) over successive cycles; see Figure 8c.^6^ During each read interval, the current exhibited transient behavior, likely due to redistribution and equilibration of carriers in the device following each abrupt voltage step.^47,58^ As a result, average read current, i.e., *I*_Read_ (avg.), was calculated and plotted for cycles 10, 20, and 30; see Figure 8c, solid orange symbols. In all cycles, the state-to-state conductance change (rise in average read current) decreased due to progressive trap filling, as expected. The ability to write/read multiple distinct conductance states shows the promise of these analog-type MT mesh memristive devices for neuromorphic computing applications.^6^

To further probe the temporal characteristics of written conductance states, states were successively created by using write pulses and periodically read over 150 s. Specifically, a write voltage pulse of +6 V (30 s) was applied to the device, followed by ten consecutive read pulses of +2 V (5 s) interleaved with 0 V (10 s) pulses; see Figure 8d. This process was repeated to create three conductance states. Read currents were plotted as black traces with average read currents, *I*_Read_ (avg.), displayed as solid orange symbols. During the first 60 s after writing a conductance state, the data exhibited initial decreases in read current with subsequent stabilization of current to a plateau value for the remainder of each measurement time window. Notably, this plateau current level was the *same* for each conductance state, indicating “forgetting” of the previously written state. Such volatile behavior mimics the transience of short-term memory in the brain, consistent with “the forgetting curve,” developed since Ebbinghaus’ pioneering investigations of forgetting in 1885, which hypothesizes how a memory fades over time if no attempt is made to retain it.^59^ In addition, in the context of ANNs, the observed biomimetic behavior, i.e., a combination of synaptic plasticity and fading memory, may enable these devices to attain a cognitive capability.^4^

### 3.5 Synaptic Emulation

In the brain, a synapse acts like a two-terminal device, with the synaptic weight, i.e., the connection strength between the participating neurons, being dynamically modifiable in a virtually analog fashion. Synaptic weights can be altered, depending on the history of synapse activity, a property known as synaptic plasticity. Synaptic plasticity provides the bedrock for information processing as well as learning and memory. Information processing involves various modes of STP, including PPF (also known as neural facilitation) and paired-pulse depression (PPD), allowing synapses to perform critical computational functions in neural circuits.^60^ Learning and memory involve LTP, which is characterized by long-lasting, activity-dependent changes in synaptic transmission and bidirectional modification of synaptic weight by potentiation (long-term potentiation) or depression (long-term depression). Synaptic plasticity strongly depends on the temporal correlation of spike signals, i.e., spiking rate, or on the time interval between such stimuli, referred to as SRDP.^3^ For example, PPF permits enhancement in the amplitude of the second of two rapidly evoked EPSCs, with a shorter time interval inducing a larger postsynaptic response.^61^ Facilitation can also be induced by brief, high-frequency trains of action potential stimuli, a phenomenon known as PTP, which also exhibits temporal dependence.^61^ In neuromorphic computing, synaptic plasticity may manifest as pulse rate-dependent plasticity (PRDP), pulse amplitude-dependent plasticity (PADP), or pulse duration-dependent plasticity (PDDP).^3,9^

**Figure 9:**
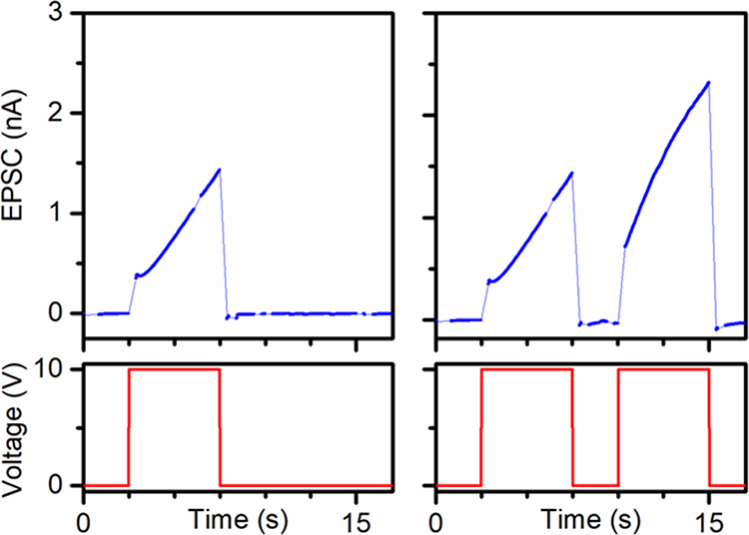
Current–time traces obtained for a SQ MT mesh device during application of various square voltage waveforms: (Left) one +10 V pulse (5 s); (Right) two +10 V pulses (5 s) with a 0 V interval pulse (2.5 s).

To characterize the synaptic plasticity of SQ MT mesh devices, current–time traces were acquired while applying square voltage waveforms with different pulse parameters as follows:

To illustrate emulation of an EPSC, one +10 V pulse (5 s) was applied; see Figure 9, left.

To illustrate emulation of PPF, two +10 V pulses (5 s) with a 0 V interval pulse (2.5 s) were applied; see Figure 9, right.

**Figure 10:**
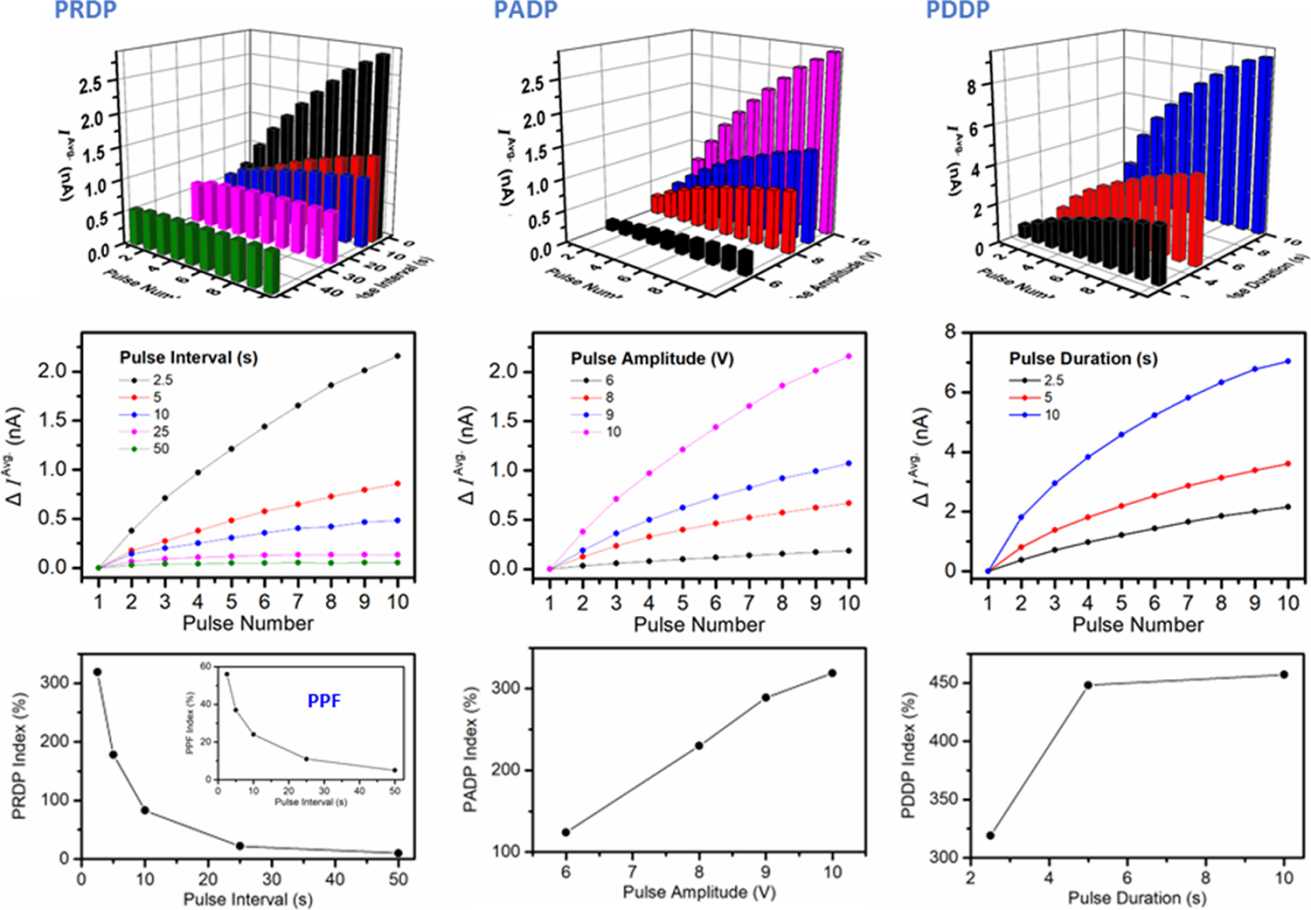
Left: (Top) Average current during each voltage pulse, calculated using the current–time data of Figure S5, versus pulse number and pulse interval; (Middle) change in *I*^Avg.^ (*n*) relative to *I*^Avg.^ (1), Δ*I*^Avg.^, versus pulse number for each pulse interval; (Bottom) pulse rate-dependent plasticity index versus pulse interval; inset: paired-pulse facilitation index vs pulse interval. Middle: analogous data acquired against pulse amplitude. Right: analogous data acquired against pulse duration.

To probe PRDP, ten +10 V pulses (2.5 s) interleaved with 0 V interval pulses (2.5, 5, 10, 25, or 50 s) were applied; see Figure S5, top row. To analyze the impact of applied voltage pulse interval on the device response, the average current, *I*^Avg.^, measured during each pulse was calculated using the current–time trace data and plotted against pulse number and pulse interval; see Figure 10, left column. By normalizing *I*^Avg.^ of subsequent pulses to that of the first pulse, the relative change in device conductance was estimated as the change in average current, Δ*I*^Avg.^, for each pulse, and Δ*I*^Avg.^ was plotted against pulse number for each pulse interval experiment. On this basis, Δ*I*^Avg.^ going from pulse 1 (first pulse) to pulse 10 (last pulse) was estimated as 2.16 nA (2.5 s pulse interval), 0.86 nA (5 s pulse interval), 0.48 nA (10 s pulse interval), 0.13 nA (25 s pulse interval), and 0.05 nA (50 s pulse interval), indicating emulation of PRDP. Also, the PRDP index, given as ([*I*^Avg.^ (10) – *I*^Avg.^ (1)]/*I*^Avg.^ (1)) × 100, was plotted, confirming that shorter pulse intervals resulted in a larger change in device conductance (synaptic weight), emulating synaptic PRDP, i.e., the temporal dependence of the synaptic weight update process. Further, Δ*I*^Avg.^ going from pulse 1 to pulse 2 was estimated as 0.37 nA (2.5 s pulse interval), 0.18 nA (5 s pulse interval), 0.14 nA (10 s pulse interval), 0.07 nA (25 s pulse interval), and 0.03 nA (50 s pulse interval), indicating emulation of PPF. The PPF index, ([*I*^Avg.^ (2) – *I*^Avg.^ (1)]/*I*^Avg.^ (1)) × 100, was plotted, confirming that shorter intervals between the two applied pulses resulted in a larger change in device conductance, emulating PPF, whereby the amplitude of an EPSC evoked by a pulse stimulus is increased when that pulse closely follows a prior pulse; see Figure 10, left column, inset.

To probe PADP, ten +6, +8, +9, or +10 V pulses (2.5 s) interleaved with 0 V interval pulses (2.5 s) were applied; see Figure S5, middle row. The average current, *I*^Avg.^, measured during each pulse was calculated using the current–time trace data and plotted against pulse number and pulse amplitude; see Figure 10, middle column. By normalizing *I*^Avg.^ of subsequent pulses to that of the first pulse, the relative change in device conductance was estimated as the change in average current, Δ*I*^Avg.^, for each pulse, and Δ*I*^Avg.^ was plotted against pulse number for each pulse amplitude experiment. The Δ*I*^Avg.^ going from pulse 1 (first pulse) to pulse 10 (last pulse) was estimated as 0.18 nA (+6 V pulse amplitude), 0.67 nA (+8 V pulse amplitude), 1.07 nA (+9 V pulse amplitude), and 2.16 nA (+10 V pulse amplitude), demonstrating emulation of PADP. Then, the PADP index was plotted, confirming that higher pulse amplitudes resulted in a larger change in device conductance (synaptic weight).

To probe PDDP, ten +10 V pulses (2.5, 5, or 10 s) interleaved with 0 V interval pulses (2.5 s) were applied; see Figure S5, bottom row. Again, the average current, *I*^Avg.^, measured during each pulse was calculated using the current–time trace data and plotted against pulse number and pulse duration; see Figure 10, right column. Then, the relative change in device conductance was estimated (as above) as the change in average current, Δ*I*^Avg.^, for each pulse and plotted against pulse number for each pulse duration experiment. The Δ*I*^Avg.^ going from pulse 1 (first pulse) to pulse 10 (last pulse) was estimated as 2.16 nA (2.5 s pulse duration), 3.61 nA (5 s pulse duration), and 7.04 nA (10 s pulse duration), indicating PDDP. The PDDP index was plotted, demonstrating that longer pulse durations resulted in a larger change in device conductance (synaptic weight).

Overall, these data indicate that SQ MT mesh devices mimic brainlike and synaptic functionality in important ways: first, the observed voltage PRDP is remarkably like synaptic plasticity, whereby synaptic weight can be modulated by consecutive spikes (memorization events). Second, increasing voltage pulse amplitudes and durations resulted in larger relative conductance increases (changes in synaptic weight), features that closely resemble the nonlinear transmission behavior of neural synapses and that enabled analog computing.

In the domain of neuromorphic computing, a large dynamic range (i.e., the current ratio of the highest to lowest conductance states) is needed to provide access to many conductance states. Also, conductance values, which emulate synaptic weights, should ideally increase (potentiation) or decrease (depression) in a linear manner according to update spikes.^4^ These are necessary features, as learning algorithms of hardware neural networks assume that *N* increments in conductance followed by *N* decrements in conductance return a synaptic device to its initial conductance state. Unfortunately, most artificial synapses do not faithfully follow this ideal and instead often exhibit rapid changes in conductance during the initial spike/pulse application process before reaching subsequent saturation.

**Figure 11:**
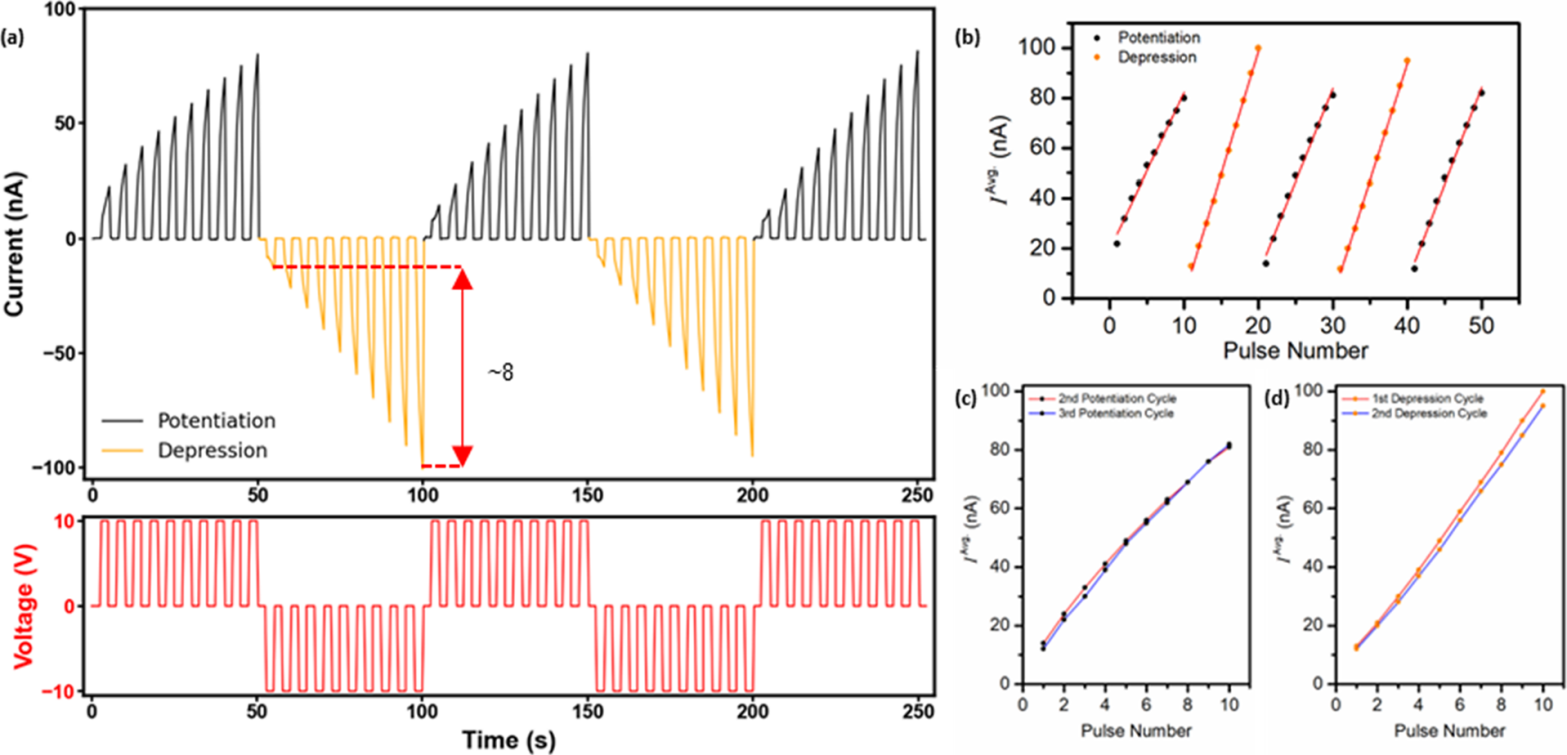
(a) Current–time trace obtained for an SQ MT mesh device during application of a square voltage waveform: ten +10 V potentiation pulses (2.5 s) interleaved with 0 V pulses (2.5 s), followed by an analogous trace consisting of ten −10 V depression pulses (2.5 s) interleaved with 0 V pulses (2.5 s), with the process repeated to a total of five cycles. (b) Average current, *I*^Avg.^, during each voltage pulse, calculated using the current–time data of (a), versus pulse number, with each potentiation and depression cycle fitted by linear regression (*R*^2^ > 0.98). (c) *I*^Avg.^ of the second and third potentiation cycles versus pulse number for each cycle. (d) *I*^Avg.^ of the first and second depression cycles versus pulse number for each cycle.

In this context, to probe potentiation and depression phenomena in SQ MT mesh devices, a square voltage waveform was applied to a device as follows: ten +10 V potentiation pulses (2.5 s) interleaved with 0 V pulses (2.5 s), followed by an analogous trace consisting of ten −10 V depression pulses (2.5 s) interleaved with 0 V pulses (2.5 s), with the process repeated to a total of five cycles; see Figure 11. This approach permitted the investigation of neuromorphic device performance metrics such as dynamic range, number of conductance states, linearity, and symmetry. With successive positive bias voltage pulses, the device conductance incrementally increased (emulation of PTP), while application of pulses of opposite polarity emulated synaptic depression, effectively resetting the device back to its original state (evident in the symmetry of the consecutive potentiation cycles). Furthermore, the dynamic range of the device, estimated during the first depression cycle, was ca. 8, an excellent value for an analog organic memristive device; see Figure 11a, red arrow.^4,6,62^

In addition, during both potentiation and depression cycles, ten conductance states were created with no suggestion that device conductance approached saturation (i.e., no deterioration in the linearity of response), suggesting that many more conductance states were likely accessible. To investigate the linearity of each potentiation and depression cycle in detail, the average current, *I*^Avg.^, measured during each pulse was calculated using the current–time trace data and plotted against pulse number, with each potentiation and depression cycle fitted by linear regression; see Figure 11b. Excellent linearity of response (linear conductance tuning) was apparent during each cycle, as illustrated by linear regressions (*R*^2^ > 0.98). Also, the average currents of the second and third potentiation cycles were plotted against pulse number for each cycle, as were, for comparison, those of the first and second depression cycles; see Figure 11c,d, respectively. The symmetric conductance tuning of the positive and negative pulse cycles was consistent with unipolar (hole-only) charge transport in these devices.

Taken together, the features of (i) a large dynamic range, (ii) access to multiple conductance states, (iii) linear, symmetric conductance tuning, and (iv) emulation of synaptic potentiation and depression phenomena combine to make this SQ MT mesh artificial e-synapse device an attractive candidate for neuromorphic computing applications.

Finally, in the psychological sciences, memory is often categorized as either short-term (STM) or long-term (LTM) associated, in neuroscience, with STP and LTP, respectively.^12,63^ Transition from STM to LTM requires changes in the brain that help protect memory from either disruptive interference (caused by injury or disease) or competing stimuli. This time-dependent process, whereby an experience achieves a permanent record in memory, is termed consolidation. At the cellular level, existing synapses can be strengthened or new synapses can form, enabling heightened communication between neurons. The psychological multistore model of Atkinson and Shiffrin states that STM can transition to LTM by rehearsal or repetition.^63^

**Figure 12:**
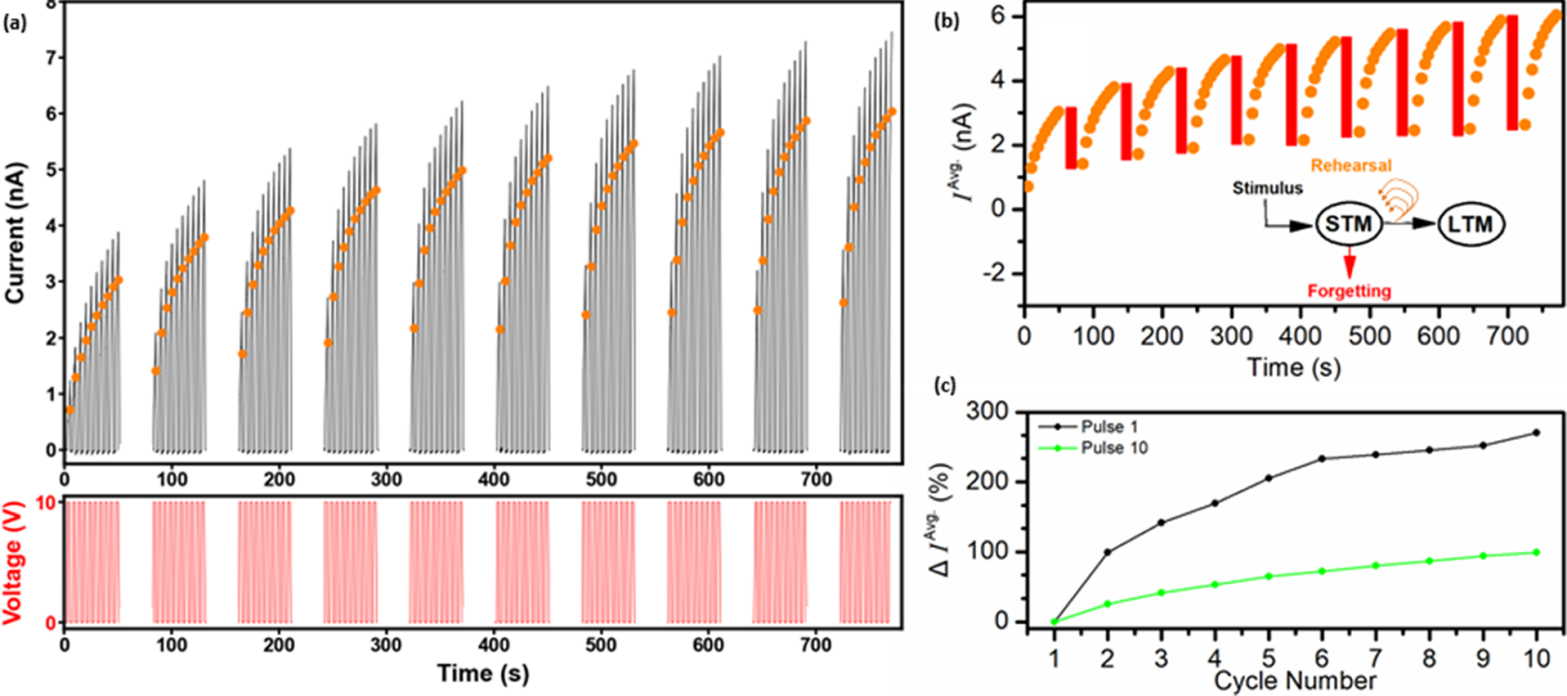
(a) Current–time trace obtained for an SQ MT mesh device during application of a square voltage waveform: ten +10 V pulses (2.5 s) interleaved with 0 V pulses (2.5 s), followed by a pause (30 s, open circuit), with the process repeated to a total of ten cycles. Average current, *I*^Avg.^, during each voltage pulse is plotted as solid orange symbols. (b) *I*^Avg.^ during each voltage pulse, calculated using the current–time data of (a), versus time, with the 30 s pause represented by red rectangles; inset: psychological model of memorization and forgetting. (c) Change in *I*^Avg.^ of Pulse 1 and Pulse 10 in each cycle, Δ*I*^Avg.^ (%), versus cycle number.

To investigate whether such learning behavior may be achieved in a SQ MT mesh device by consolidation or rehearsal (repeated application of stimuli), a square voltage waveform was applied: ten +10 V pulses (2.5 s) interleaved with 0 V pulses (2.5 s), followed by a pause (30 s, open circuit), with the process repeated to a total of ten cycles; see Figure 12a. The average current, *I*^Avg.^, during each voltage pulse was calculated and plotted; see Figure 12a,b, solid orange symbols. As expected, within a cycle, the average current (device conductance) increased with successive pulses from pulse 1 (first pulse) to pulse 10 (last pulse). Also, the average current for pulse 1 of a cycle was always lower than that for pulse 10 of the prior cycle, i.e., conductance decayed during each open-circuit pause, indicating volatile behavior.^12^ In addition, from cycle to cycle, values of the average current for corresponding pulses increased, demonstrating emulation of learning behavior, i.e., a PTP-induced transition from STM to LTM following a high-frequency train of stimuli. Further, by normalizing the pulse 1 *I*^Avg.^ values of subsequent cycles to the pulse 1 *I*^Avg.^ value of the first cycle, the relative change in device conductance was estimated as the change in average pulse 1 current, Δ*I*^Avg.^, from cycle to cycle. Then, Δ*I*^Avg.^ was plotted against cycle number; see Figure 12c. This procedure was also applied to the pulse 10 *I*^Avg.^ values. By inspection, Δ*I*^Avg.^ values for pulse 1 and for pulse 10 were observed to increase from cycle to cycle with cumulative increases of ca. 300 and 100%, respectively.

This consolidation of device conductance emulated human memory and demonstrated that repetition rehearsal was evidently an appropriate method for increasing memory strength; see the schematic in Figure 12b, inset. Thus, the e-synapse properties of the SQ MT mesh devices potentially extend beyond emulation of synaptic behavior and offer utility in, e.g., psychology, for implementing models of human memory in the brain.^13^

## 4 Conclusions

In this work, MTs based on SQ were prepared by EISA from a solvent:nonsolvent mixture. The MTs were approximately 2 μm in diameter (with aspect ratios of 10–130) and P-XRD analysis indicated the presence of monoclinic and orthorhombic polymorphs. Optical measurements gave values for the onset of optical absorption and for the optical energy gap consistent with the monoclinic phase, suggesting that this was the dominant polymorph, while Urbach analysis indicated the presence of energetic disorder. Photoelectron spectra gave a value for the HOMO energy, and spectral deconvolution suggested atomic environments consistent with a quinoidic structure of the anilino rings associated with the betaine-type arrangement obtained from single crystal X-ray diffraction data.

Given the favorable energetic alignment between the work function of Au and the HOMO energy of the squaraine, symmetric, unipolar (hole-only) metal–insulator–metal-type devices were formed by EISA of MT meshes on interdigitated Au electrodes. The DC *I–V* characteristics acquired from such devices displayed pinched hysteretic *I–V* loops, indicative of memristive behavior. Analysis of the *I–V* data indicated an Ohmic transport at low bias with carrier extraction facilitated by thermionic emission. At high biases, the devices exhibited trap-limited space-charge-limited conduction in the presence of traps distributed in energy, which was enhanced by a Poole-Frenkel effect, with carrier extraction facilitated by FN tunneling. Taken together, the data indicated purely electronic conduction. Interestingly, *I–V* hysteresis was attenuated at smaller voltage windows, suggesting that carrier trapping/detrapping underpinned the hysteretic current. During voltage sweeps applied to MT mesh devices using triangular waveforms, *I–V* hysteresis and analog RS (memristive device) functionality were observed. Device conductance could be gradually increased sweep by sweep, giving conductance tuning through distinct states with wait time or voltage-erase options consistent with trap filling/emptying effects. Also, using square waveforms, repeated erase-write-read of multiple distinct conductance states was demonstrated, highlighting the promise of these MT mesh-based analog memristive devices for neuromorphic computing applications.

By waveform design, volatile conductance states could also be written so that successive conductance states exhibited identical current levels, indicating forgetting of previously written states and thereby mimicking the forgetting curve. This combination of synaptic plasticity and fading memory illustrated the potential of these devices for incorporation into ANNs, with the possibility of attaining cognitive capability. In addition, an organic MT mesh memristive device on a PET substrate exhibited flexibility under a bending test, suggesting potential utility for the development of green, wearable electronics. Finally, advanced synaptic functions, i.e., EPSCs, PPF, pulse-dependent plasticity, and a transition from short- to long-term memory driven by PTP, were demonstrated. In conclusion, operating on the principle of purely electronic RS, these novel organic semiconductor MT mesh devices provide an attractive combination of large dynamic range, access to multiple conductance states, linear and symmetric conductance tuning, and biorealistic synaptic emulation.

## Supporting Information Available

Crystal data for TCD1539 (CIF)
Crystal data for TCD1651 (CIF)
Single crystal analysis; effect of [SQ] during preparation; DC measurements under illumination; and pulse-dependent plasticity (raw data) (PDF)

## Supplementary Material

am3c13735_si_001.cif

am3c13735_si_002.cif
