# Supplementary material for: Volatile Memristive Devices with Analog Resistance Switching Based on Self-Assembled Squaraine Microtubes as Synaptic Emulators
Source: ACS Appl Mater Interfaces. 2024 Jan 4;16(2):2539–53. doi: 10.1021/acsami.3c13735 (PMC10797587; doi:10.1021/acsami.3c13735)
Supplement: Supplementary file 3 — am3c13735_si_003.pdf [file am3c13735_si_003.pdf]

Supporting Information

for

Volatile Memristive Devices with Analogue Resistance Switching Based on Self-assembled Squaraine Microtubes as Synaptic Emulators

*Karl Griffin and Gareth Redmond**

*gareth.redmond@ucd.ie

School of Chemistry, University College Dublin, Belfield, Dublin 4, Ireland

SI.I Single Crystal Analysis

SI.II Effect of [SQ] During Preparation

SI.III DC Measurements Under Illumination

SI.IV Pulse-dependent Plasticity (Raw Data)

SI.I Single Crystal Analysis

Methods. For the monoclinic phase, a green block-like crystal specimen of $C_{32}H_{44}N_2O_6$, approximate dimensions 0.080 mm × 0.180 mm × 0.190 mm, was used for the X-ray crystallographic analysis. The X-ray intensity data were measured (λ = 0.71073 Å) at 100 K on a Bruker D8 Quest ECO with an Oxford Cryostream low temperature device using a MiTeGen micromount. Bruker APEX software was used to correct for Lorentz and polarisation effects. A total of 255 frames were collected. The total exposure time was 3.54 hours. The integration of the data using a monoclinic unit cell yielded a total of 15017 reflections to a maximum θ angle of 26.56° (0.79 Å resolution), of which 3039 were independent (average redundancy 4.941, completeness = 99.5%, R_{int} = 13.49%, R_{sig} = 9.52%) and 1804 (59.36%) were greater than 2σ(F^2). The final cell constants of $\underline{a}$ = 6.1893(8) Å, $\underline{b}$ = 16.4482(19) Å, $\underline{c}$ = 14.3989(18) Å, β = 92.351(5)°, volume = 1464.6.5(3) Å^3, are based upon the refinement of the XYZ-centroids of 3548 reflections above 20 σ(I) with 5.705° < 2θ < 52.48°. The calculated minimum and maximum transmission coefficients (based on crystal size) are 0.9840 and 0.9930. The structure was solved with the SHELXT structure solution program using Intrinsic Phasing and refined with the SHELXL refinement package using Least Squares minimisation with Olex2, using the space group $P2_1/n$, with Z = 2 for the formula unit, $C_{32}H_{44}N_2O_6$. The final anisotropic full-matrix least-squares refinement on F^2 with 193 variables converged at $R1$ = 5.77%, for the observed data and $wR2$ = 13.03% for all data. The goodness-of-fit was 1.047. The largest peak in the final difference electron density synthesis was 0.264 $e^-Å^{-3}$ and the largest hole was -0.232 $e^-Å^{-3}$ with an RMS deviation of 0.061 $e^-Å^{-3}$. On the basis of the final model, the calculated density was 1.253 g cm^{-3} and $F(000)$, 596 e^-. The single crystal structure data were visualised and analysed with Mercury (2020.1 CSD Release), available free of charge from www.ccdc.cam.ac.uk/mercury/.

For the orthorhombic phase, a metallic green rod-like crystal specimen of $C_{32}H_{44}N_2O_6$, approximate dimensions 0.080 mm × 0.080 mm × 0.180 mm, was used for the X-ray crystallographic analysis. The X-ray intensity data were measured (λ = 0.71073 Å) at 100 K on a Bruker D8 Quest ECO with an Oxford Cryostream low temperature device using a MiTeGen micromount. Bruker APEX software was used to correct for Lorentz and polarisation effects. A total of 1318 frames were collected. The total exposure time was 18.31 hours. The integration of the data using an orthorhombic unit cell yielded a total of 61626 reflections to a maximum θ angle of 29.62° (0.72 Å resolution), of which 4154 were independent (average redundancy 14.835, completeness = 99.7%, R_{int} = 11.84%, R_{sig} = 7.41%) and 2355 (56.69%) were greater than 2σ(F^2). The final cell constants of $\underline{a}$ = 15.0597(7) Å, $\underline{b}$ = 18.1899(9) Å, $\underline{c}$ = 10.7693(5) Å, volume = 2950.1(2) $Å^3$, are based upon the refinement of the XYZ-centroids of 5894 reflections above 20 σ(I) with 5.160° < 2θ < 58.97°. The calculated minimum and maximum transmission coefficients (based on crystal size) are 0.9850 and 0.9930. The structure was solved with the SHELXT structure solution program using Intrinsic Phasing and refined with the SHELXL refinement package using Least Squares minimisation with Olex2, using the space group *P*bcn, with Z = 4 for the formula unit, $C_{32}H_{44}N_2O_6$. The final anisotropic full-matrix least-squares refinement on F^2 with 218 variables converged at $R1$ = 4.84%, for the observed data and $wR2$ = 11.17% for all data. The goodness-of-fit was 0.940. The largest peak in the final difference electron density synthesis was 0.333 $e^-Å^{-3}$ and the largest hole was -0.198 $e^-Å^{-3}$ with an RMS deviation of 0.049 $e^-Å^{-3}$. On the basis of the final model, the calculated density was 1.244 g cm^{-3} and $F(000)$, 1192 e^-. The single crystal structure data were visualised and analysed with Mercury (2020.1 CSD Release).

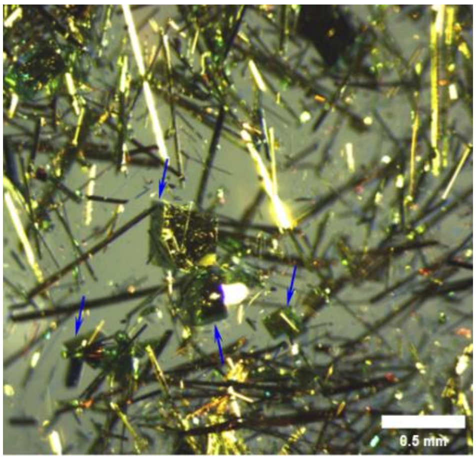

Figure S1.1. Reflected light optical microscopy image of SQ single crystals. The monoclinic blocks are indicated by blue arrows while the orthorhombic rods are also visible.

SI.II Effect of [SQ] During Preparation

Methods and Outcomes. Additional formulations were screened. Specifically, a 1 mL aliquot of a 0.1 mg mL^{-1} SQ/DCM solution (ca. 10^{-4} M) was added to a series of 5 mL H_2O:EtOH solutions (1:3, 2:3, 1:1 and 3:2 (v/v)) under vigorous stirring. After 3 min, stirring was ceased, and the mixture was allowed to stand for 1 h. For samples with 3:2 and 1:1 H_2O:EtOH, phase separation to an aqueous layer (top) and an organic layer (bottom) occurred in each vial. Aliquots from the organic layer were transferred from each vial onto appropriate inspection substrates via pipette aspiration where precipitation of solid SQ material subsequently occurred during drying. For the 2:3 and 1:3 H_2O:EtOH samples, no distinct phase separation occurred, and mild precipitation was observed. Consequently, a sample of precipitated solid material plus liquid was transferred from each vial onto an appropriate inspection substrate via pipette aspiration.

Reflected light optical microscopy images of all samples are shown in Figure SI.2. For 1:3 H_2O:EtOH, plate-like morphologies were observed in the precipitate. For 2:3 H_2O:EtOH, mixed rod- and plate-like morphologies were observed in the precipitate. In contrast, for 1:1

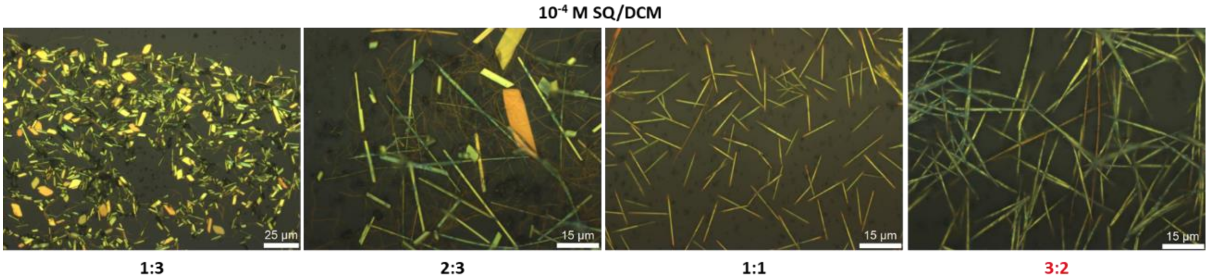

Figure SI.2. Reflected light optical microscopy images of materials obtained during non-solvent (H_2O:EtOH) screening in high aspect ratio fiber formation at lower SQ concentration; each H_2O:EtOH ratio (v/v) is indicated.

and 3:2 H_2O:EtOH, higher aspect ratio SQ morphologies, with amorphous SQ material, were apparent. Importantly, the 3:2 H_2O:EtOH sample contained abundant SQ fibers, likely formed successfully by in situ evaporation-induced self-assembly (EISA) with a low background of amorphous material. Extensive optical imaging of the 3:2 H_2O:EtOH SQ sample confirmed that this material comprised a dense mesh of randomly distributed one-dimensional SQ fibers with a gold colour. SEM images of fibers prepared using 1.0/0.1 mg mL^{-1} SQ/DCM solutions (ca. $10^{-3}/10^{-4}$ M) are shown in Figure SI.3. The square profile of the structures is apparent in both images; at lower [SQ], the structures were smaller. Analysis of image data gave (ca. 10^{-3} M) an average diameter of circa 1.9 µm ± 0.9 µm and (ca. 10^{-4} M) an average diameter of circa 0.8 µm ± 0.2 µm.

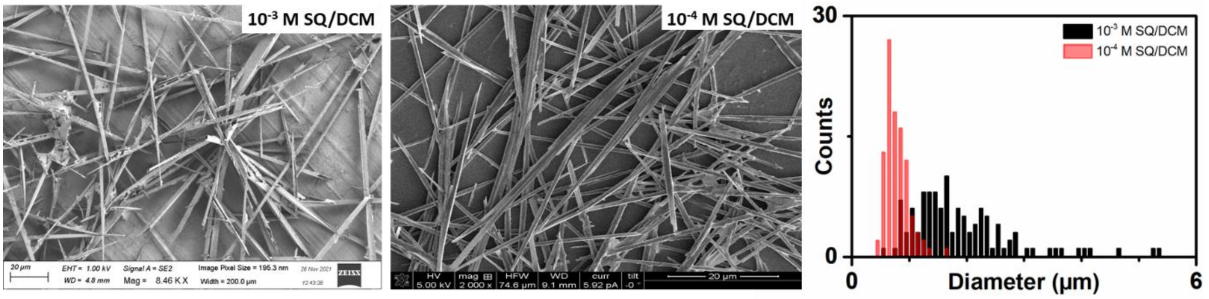

Figure SI.3. Comparative SEM images and diameter histograms for the 3:2 H_2O:EtOH SQ samples obtained from 1.0/0.1 mg mL^{-1} SQ/DCM solutions (ca. $10^{-3}/10^{-4}$ M).

SI.III DC Measurements Under Illumination

DC *I-V* characteristics acquired for a SQ MT mesh device while under illumination (low pressure mercury vapour discharge (broadband) lamp; ca. 18 μW at 532 nm) are plotted in Figure SI.4. Comparison of the light data with dark data acquired from the same device (Figure 6(b)) indicated a significant increase in current with illumination, suggesting a photoconductor-type response by light-induced carrier generation, and consistent with the noted panchromatic absorbance of the SQ MTs. Device illumination did not appreciably affect the shape of the *I-V* loop consistent with similar carrier transport and injection/extraction mechanisms under illumination and in the dark. Specifically, in the light, low- and high-bias regions of the *I-V* curves (outward) were also observed and correlated with TE-enabled Ohmic transport and FN-enabled TL-SCLC with PF enhancement, respectively. Importantly, SEM image data for the device acquired following these measurements exhibited no gross signs of degradation, indicating promising measurement- and photo-stability of the SQ MTs. Demonstration of a pronounced photo-response in these devices points the way to consideration of future materials/device design formats that utilise both electrical and optical input signals.

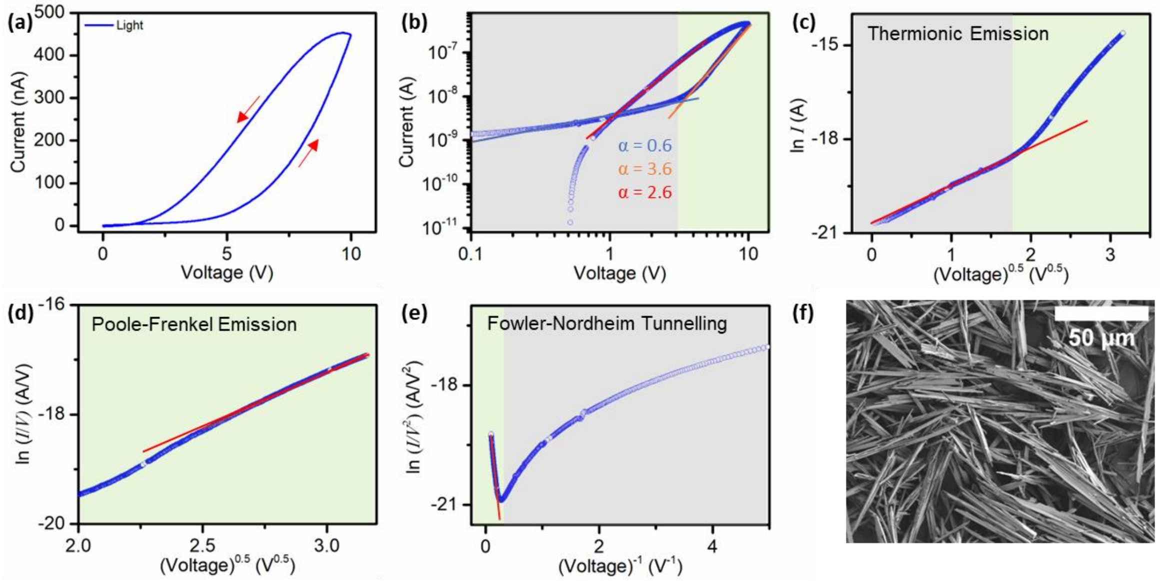

Figure SI.4. (a) *I-V* loop acquired in the light for a SQ MT mesh device on a linear scale. (b) The data plotted on a double logarithmic scale; slopes (α) of linear regions obtained by linear regression fits (R^2 > 0.99). (c) TE fit at low bias. (d) PF fit at high bias. (e) FN fit at high bias. (f) SEM image of the MTs post-measurement.

SI.IV Pulse-dependent Plasticity (Raw Data)

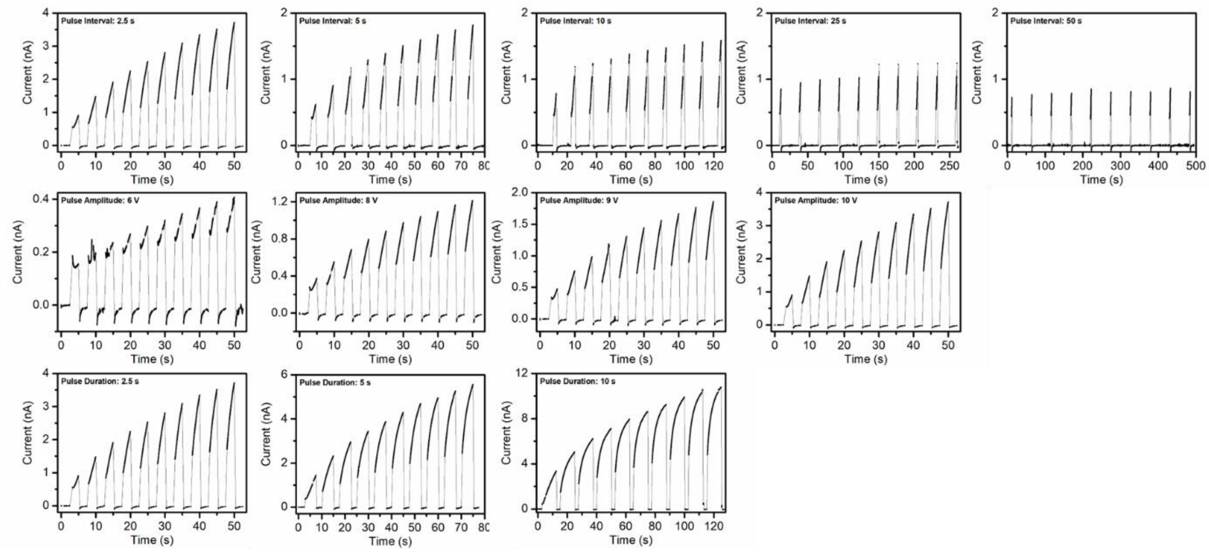

Figure SI.5: Current-time traces obtained for a SQ MT mesh device during application of various square voltage waveforms: (Top) Ten +10 V pulses (2.5 s) interleaved with 0 V interval pulses (2.5 s, 5 s, 10 s, 25 s or 50 s); (Middle) Ten +6 V, +8 V, +9 V or +10 V pulses (2.5 s) interleaved with 0 V interval pulses (2.5 s); (Bottom) Ten +10 V pulses (2.5 s, 5 s or 10 s) interleaved with 0 V interval pulses (2.5 s).
